# Systematic detection of predictive gene sets by semantics-based selection

**DOI:** 10.1186/s12859-026-06570-5

**Published:** 2026-08-11

**Authors:** Peter Eckhardt-Bellmann, Nahla A. Taha, Silke D. Werle, Johann M. Kraus, Nensi Ikonomi, Hans A. Kestler

**Affiliations:** 1 Institute of Medical Systems Biology, Ulm University, Albert-Einstein-Allee 11, 89081 Ulm, Baden-Württemberg Germany; 2 Leibniz Institute on Aging, Fritz Lipmann Institute, Beutenbergstraße 11, 07745 Jena, Thuringia Germany

**Keywords:** Gene ontology, Gene selection, Gene functions, GO terms

## Abstract

**Background:**

The Gene Ontology (GO) is a public resource that describes gene functions and characteristics through a structured vocabulary of standardised terms. It currently contains annotations for over 1.5 million gene products, each linked to one or more GO terms. In this study, we propose integrating this GO-based semantic structure into machine learning systems for medical diagnostics. This approach serves a dual purpose: first, to prioritise genes that are semantically relevant to a given clinical task, thereby refining model input; and second, to enable the analysis of biologically predefined gene sets, which may reveal novel mechanisms underlying disease.

**Results:**

Evaluated across 16 benchmark data sets spanning diverse medical domains, our GO term-informed gene selection method generally outperformed models trained on full gene sets. Further analysis of individual GO terms not only enhanced classification performance but also identified high-performing, task-specific gene subsets that were overlooked during initial gene selection.

**Conclusion:**

Our findings demonstrate that Gene Ontology can be effectively leveraged for semantics-aware gene selection in clinical machine learning. Moreover, systematically evaluating individual GO terms offers a scalable strategy to uncover new, testable biological hypotheses–revealing gene functions that might otherwise remain hidden when examining only broadly selected gene combinations.

## Background

Artificial intelligence (AI) is a popular tool, which can be utilised in various domains, such as in agriculture or the automotive and film industries, amongst many others, including the healthcare sector [1]. In healthcare, intelligent systems can be used to assist nurses and physicians with different medical tasks, for instance with the detection of pain [2] or diagnosis of diseases [3]. Depending on the type of the disease, diagnoses can be based on all kind of different inputs, ranging from a simple description of the symptoms by the patient, over computed tomography (CT) and magnetic resonance imaging (MRI) scans, up to urine, stool, blood or tissue samples. In recent years, advanced molecular high-throughput techniques have increasingly supported the classical diagnostic methods discussed above. Different types of omics data can be collected, including gene expression data. In general, gene expression data are high-dimensional, which, paired with a low number of data points, leads to one of the main challenges in medical settings [4]. That is, put simply: too few samples (subjects/participants/patients), too many features (measurements, e.g. gene expression values).

High-dimensional data often contains redundant information, called noise. Machine learning (ML) models that are trained in combination with a low number of high-dimensional samples tend to face the issue of overfitting, which is defined as the lack of the ability to generalise. More precisely, an overfitted model might perform well on data it was trained with, however, a significant drop in performance is observed when the model is evaluated on samples unseen during the training phase. One solution to overcome this obstacle is to drastically reduce the high dimensionality by applying some feature selection (FS) techniques [5–7], which is especially common in the course of microarray, bulk and single-cell RNA sequencing data analysis [8–11]. Due to their versatility, FS approaches can be grouped into different categories, e.g. into the typical three categories filter, wrapper, and embedded type FS, or simply into the binary categories univariate and multivariate type FS.

Despite the algorithmic differences of existing FS methods, which lead to different disadvantages and benefits, they all have the common ground of ignoring the inherent meanings of features. Thus, our idea is to include semantics into the process of FS of gene expression patterns. The main motivation is that, depending on the task at hand, certain genes are expected to be less or more important than others, leading to better classification performance [12, 13]. For example, specific gene sets that can be used as biomarkers for the detection of one cancer entity (e.g. prostate cancer) most likely differ from those effectively used in the detection of another cancer entity (e.g. breast cancer) [14]. Therefore, we propose to individually incorporate task-specific semantic knowledge for the selection of relevant genes. Simultaneously, we want our gene selection approach to be applicable even in the absence of human experts. This can be achieved by retrieving expert knowledge from publicly available knowledge bases.

In this work, we propose making use of the publicly available Gene Ontology Resource (GOR) [15]. The GOR is "the world’s largest source of information on the functions of genes".^1^ The genes in the GOR are grouped by their functions, providing a link between gene sets and semantic labels. These function-specific gene groups, called GO terms, constitute the central focus of our study. Using the semantic knowledge encoded in these GO terms, the main aim of this work is to develop and validate gene selection approaches that identify classification task-relevant gene subsets, leading to a significant reduction of the initially high-dimensional feature space while maintaining or even improving classification performance. We pursue this aim through the following two main contributions.

1. As our first contribution, we introduce our semantics-specific gene selection approach in which we screen for task-relevant subsets of genes by filtering out GO terms adequate to the corresponding task and/or type of samples.
2. As our second contribution, we show that the GO term-specific structure can be exploited to explore the whole gene space to unveil undiscovered connections between diseases and gene expression profiles.

The remainder of this work is organised as follows. The motivation for our study is presented in “Motivation” section, including the summary of two essential related works. In “Methodology” section, we present the general composition of GO terms based on a specific example and subsequently introduce our proposed semantics-based gene selection approach, followed by the applied evaluation metrics. A brief overview of the analysed data sets and our data preprocessing steps are provided in “Analysed data sets and data preprocessing” section, which is followed by the specification of our applied experimental settings in “Evaluation protocol” section. We present and discuss the outcomes of our proposed gene selection approach in “Experiments: keywords-based gene selection” section. Additional experiments are conducted and discussed in “Experiments: comparison against single GO terms” section, in which we analyse the task-specific prediction effectiveness of all individual GO terms. The specifics of our proposed frameworks and their validation are discussed in detail in “Discussion” section. Finally, in “Conclusion” section, we conclude this article and state our ideas for future research directions.

Note that throughout this entire work, we will use the terms gene(s) and gene product(s) interchangeably.

## Motivation

Recent studies have shown the effective role of using Gene Ontology (GO) information in combination with machine learning to better understand gene functions and improve disease classification. For example, Asif et al. [16] used GO-based gene functional similarity scores as input features for machine learning classifiers such as Support Vector Machines [17, 18] and Random Forests [19]. Their study demonstrated that GO-driven features resulted in better classification performance than raw gene expression data, emphasising the biological relevance of GO-based dimensionality reduction. Similarly, Pritykin et al. [20] introduced a computational approach to identify multifunctional genes at the genome-wide level using functional annotations, demonstrating that genes involved in multiple biological processes possess distinct properties and are significantly more likely to be involved in human disorders.

While these studies have focused on either measuring gene similarity based on GO annotations [16] or identifying multifunctional genes using GO functional annotations [20], our approach takes a different direction. Building on these studies, which have established strong associations between diseases and GO terms, our work approaches the problem differently by benefiting from these associations. Using the specific GO term/gene set relationships, we aim to develop a semantics-based gene selection methodology. Our approach reduces the high-dimensional gene sets by focusing on the genes that are most likely to be related to the specific diseases of the current classification tasks.

## Methodology

In this section, we introduce our proposed semantics-based gene selection approach, followed by a brief description of the applied evaluation metrics.

### Gene ontology

Gene Ontology (GO) is "a controlled vocabulary comprising descriptive terms for annotating genes" [21, p. 313]. GO terms consist of several characteristics, including the GO ID, GO term name, Ontology (**BP**: **B**iological **P**rocess, **MF**: **M**olecular **F**unction, **CC**: **C**ellular **C**omponent), and a definition, amongst others. An example of the GO term *prostate gland development* is shown in Table 1, with a focus on the first six attributes.

**Table 1:** Gene Ontology Example Entry for the GO Term *Prostate Gland Development*

Accession	GO:0030850
Name	Prostate Gland Development
Ontology	Biological Process
Synonyms	Prostate Development
Alternate IDs	None
Definition	The process whose specific outcome is the progression of the prostate gland
	over time, from its formation to the mature structure. The prostate gland is $\cdot\cdot\cdot$

Retrieved from https://amigo.geneontology.org/amigo/term/GO:0030850 (Last accessed: 09/03/26)

### Keywords-based gene selection

The idea for our proposed semantics-specific gene selection approach is based on the consideration that each disease is most likely associated with only certain groups of genes. Therefore, instead of analysing the entirely available gene expression data of a patient, we expect it to be more beneficial to focus on subsets of genes relevant to the current disease. In addition, the goal was to provide a reliable and reproducible choice of gene sets when there is a lack of human experts during the gene selection process, or as a preselection for human experts. More precisely, we wanted the gene selection process to be effective in enhancing the detection of different types of diseases (e.g. cancer, alcoholism, influenza, etc.) even when it is not possible to receive any direct semantic input from practitioners or biological experts of the corresponding medical specialisations.

To this end, we propose making use of the publicly available Gene Ontology knowledge base. The main advantage is that many genes are directly annotated to at least one GO term. For instance, the GO term *prostate gland development* (cf. Table 1) comprises a total of 16 directly annotated human genes (ALOX15B, FKBP4, GLI1, HOXA9, HOXA10, HOXA11, HOXA13, HOXB13, HOXD13, NKX3-1, PTCH1, RARA, SHH, SMARCC1, SOX9, TP63).

Based on these specific GO terms and their associated genes, we introduce a semantics-based gene selection approach, which consists of the following three steps:

i) Choose a set of keywords related to the task.
ii) Retrieve all GO terms including the keywords in the GO term name.
iii) Focus on the gene subset associated with the GO terms.

**Fig. 1:**
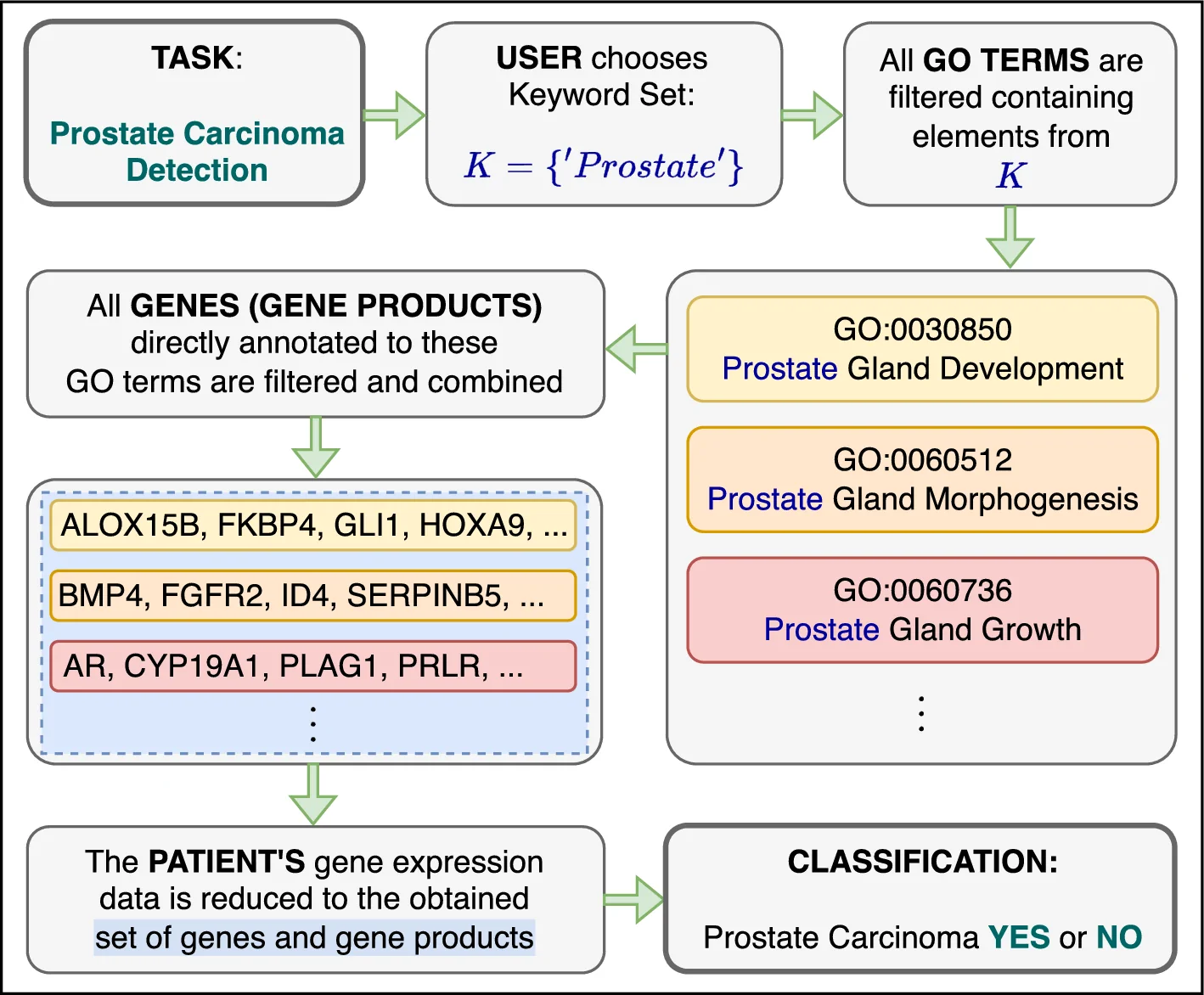
Gene Selection Pipeline. Here, an example for a prostate carcinoma detection task is depicted

As an example, let us consider the task of detecting prostate carcinoma from prostate tissue. We can simply assume that genes which are associated with the prostate constitute a helpful set of indicators for the detection of prostate cancer. Therefore, in Step i), we could choose the straightforward keyword ‘prostate’. In total, 23 GO terms include the keyword ‘prostate’ in the GO term name, e.g. *prostate gland development* (GO:0030850), *prostate gland growth* (GO:0060736), and *prostate gland morphogenesis* (GO:0060512), which would then be retrieved in Step ii). Finally, in Step iii), we would combine the sets of genes that are directly annotated to the obtained GO terms. The example is illustrated in Fig. 1.

Note that in addition to reproducibility, another advantage of selecting generic keywords, as in the example above, is the reduction of input biases by considering the least assumptions possible in generating the sets that are fed to the GO term base.

### Evaluation metrics

To evaluate the classification performance of our proposed method, in this work, we utilised the accuracy score and the macro-averaged accuracy, for the analysed balanced and imbalanced data sets, respectively.

#### Accuracy score

Accuracy is one of the most fundamental performance metrics used to evaluate classification models. It is defined as the ratio of correctly predicted instances to the total number of instances in the data set. It provides a straightforward measure of how well a classifier performs, especially when the data set is balanced [22]. The accuracy score is mathematically represented as

$$\begin{aligned} \text {Accuracy} = \frac{\text {TP} + \text {TN}}{\text {TP} + \text {TN} + \text {FP} + \text {FN}}, \end{aligned}$$

with the following definitions:

- TP (True Positives):
  The number of correctly predicted positive cases.
- TN (True Negatives):
  The number of correctly predicted negative cases.
- FP (False Positives):
  The number of negative cases incorrectly predicted as positive cases.
- FN (False Negatives):
  The number of positive cases incorrectly predicted as negative cases.

#### Macro-averaged accuracy

For imbalanced data sets, measures other than the accuracy score have to be considered to account for the class imbalance. One of the existing metrics is the macro-averaged accuracy, which is defined as

$$\begin{aligned} \text {Macro-averaged Accuracy} = \frac{1}{2} \times \left( \frac{\text {TP}}{\text {TP} + \text {FN}} + \frac{\text {TN}}{\text {TN} + \text {FP}}\right) , \end{aligned}$$

with TP, FN, TN, and FP defined as above. In the macro-averaged accuracy the class-specific accuracy values are weighted equally, ignoring the difference in the number of samples between the majority and minority class.

## Analysed data sets and data preprocessing

The characteristics of the data sets analysed in this work are summarised in Table 2. All data sets utilised in this study are publicly available from the NCBI Gene Expression Omnibus^2^ (GEO), a functional genomics data repository, and Kaggle,^3^ a data science competition platform. In this work, we include gene expression data from microarrays (miArr) and sequencing (RNA-Seq), and DNA methylation (DNAm) data. The preprocessing steps applied for each of the data types are described in the following. Note that detailed descriptions of data sets are provided in the appendix, in Appendix: Data set description.

### Data preprocessing of microarray data

For each of the microarray data sets analyzed in this work, we downloaded the raw CEL files from Gene Expression Omnibus under the GEO ID provided in Table 2. A CEL file is "a data file created by Affymetrix DNA microarray image analysis software. It contains the data extracted from ‘probes’ on an Affymetrix GeneChip" [23, p. 4]. We applied the MAS5 [24] algorithm for normalization of the raw GeneChip data. Subsequently, we mapped the GeneChip-specific probe IDs to their corresponding gene symbols (gene names) in the organism Homo sapiens. Probe IDs leading to *NA*, i.e. probe IDs not corresponding to any human gene, were discarded. Furthermore, expression values of probe IDs corresponding to the same gene were grouped and combined to one single value by the geometric mean, which is defined as

$$\begin{aligned} \text {GM}(x_{1}, \ldots , x_{m}) = \root m \of {\prod _{i=1}^{m} x_{i}}, \quad (\text {well-defined if } x_{i} > 0 \; \forall i = 1, \ldots , m). \end{aligned}$$

Applying these preprocessing steps led to a total of 22,473, 14,196, and 9,949 gene expression values for the GeneChips HG-U133_Plus_2 (data sets Ageing, Alcoholism, and Pancreatic Cancer), HG-U133A_2 (both Influenza A subtypes), and HG-U95Av2 (Prostate Cancer), respectively, as reported in Table 2.

### Data preprocessing of RNA-Seq data

For the four Kaggle data sets (Liver, Prostate, Thyroid, and Lung Cancer), as well as the Paediatric Leukaemia data set, we downloaded the preprocessed data. As preprocessing of the remaining RNA-Seq data set, Liver Disease, we applied the Counts per Million (CpM) method [25], which is defined as

$$\begin{aligned} \text {CpM}(g_{j}) = c_{j} \times \left( \sum _{i=1}^{N} c_{i} \right) ^{-1} \times 10^{6}, \end{aligned}$$

whereby $c_{j}$ denotes the count of gene $g_{j}$, and *N* denotes the total number of genes in the sample. Moreover, the genes of the Liver Disease and Paediatric Leukaemia data sets are provided as Ensembl IDs,^4^ which we mapped to the corresponding gene names (see Table 10, “Evaluation protocol” section). Values of duplicate gene names were combined by the arithmetic mean. Finally, genes that had a constant zero count across all samples were removed.

### Data preprocessing of DNAm data

For all four DNAm data sets, Tuberculosis, Prostate, Breast, and HCC, we downloaded the preprocessed data from the GEO under the GSE IDs listed in this section (cf. Table 2). For both devices, Illumina Infinium MethylationEPIC Versions 1 and 2, we additionally downloaded the corresponding mapping tables, which can be found under the GEO accession IDs, GPL21145 (Version 1) and GPL33022 (Version 2). In both mapping tables, the gene names are listed in column *UCSC_RefGene_Name*. As an initial step, we removed all IDs with missing gene names. Subsequently, we grouped the probe IDs corresponding to the same gene and combined them to one single value by computing the arithmetic average. This process led respectively to a total of 25,345 (Tuberculosis), 23,980 (HCC), 24,980 (Breast), and 25,603 (Prostate) genes.

**Table 2:** Analysed Data Sets. Top: Microarray Data. Middle: RNA-Seq Data. Bottom: DNAm Data

Data set	# Genes	Class Distribution		Class Meanings		Data Sources	
		# Class 1	# Class 2	Class 1	Class 2	GEO ID	Ref
Ageing	22,473	21	20	Below 69	Above 69	GSE53890	[26]
Pancreas	22,473	36	36	Normal	Tumour	GSE15471	[27]
Alcohol	22,473	21	21	Control	Alcoholic	GSE52553	[28]
– – – – – – – – – – – – – – – – – – – – – – – – – – – – – – – – – – – – – – – – – – – – – – – – – – – – – – – – – – – – – –							
Prostate	9,949	63	65	Normal	Tumour	GSE6608	[29]
Prostate	9,949	63	65	Normal	Tumour	GSE6606	[29]
– – – – – – – – – – – – – – – – – – – – – – – – – – – – – – – – – – – – – – – – – – – – – – – – – – – – – – – – – – – – – –							
H3N2	14,196	8	9	Asympt.	Sympt.	GSE52428	[30]
H1N1	14,196	12	11	Asympt.	Sympt.	GSE52428	[30]

Liver	20,530	50	372	Normal	Tumour	Kaggle$^{+}$	[31]
Prostate	20,530	52	498	Normal	Tumour	Kaggle$^{+}$	[31]
Thyroid	20,530	59	513	Normal	Tumour	Kaggle$^{+}$	[31]
Lung	20,530	110	989	Normal	Tumour	Kaggle$^{+}$	[31]
– – – – – – – – – – – – – – – – – – – – – – – – – – – – – – – – – – – – – – – – – – – – – – – – – – – – – – – – – – – – – –							
Liver	40,131	27	216	Normal	Disease	GSE263786	[32]
Leukaemia	35,087	8	65	Normal	Tumour	GSE227832	[33]

Tuberculosis	25,345	10	10	Absent	Active	GSE304107	[34]
Prostate	25,603	26	27	Normal	Tumour	GSE262524	[35]
Breast	24,980	113	224	Normal	Tumour	GSE225845	[36]
HCC	23,980	260	221	Control	HCC	GSE281691	[37]

$^{+}$ URL: https://www.kaggle.com/datasets/tianjiechen/tcga-rna-datasets (Last accessed: 09/03/26)

## Evaluation protocol

**Fig. 2:**
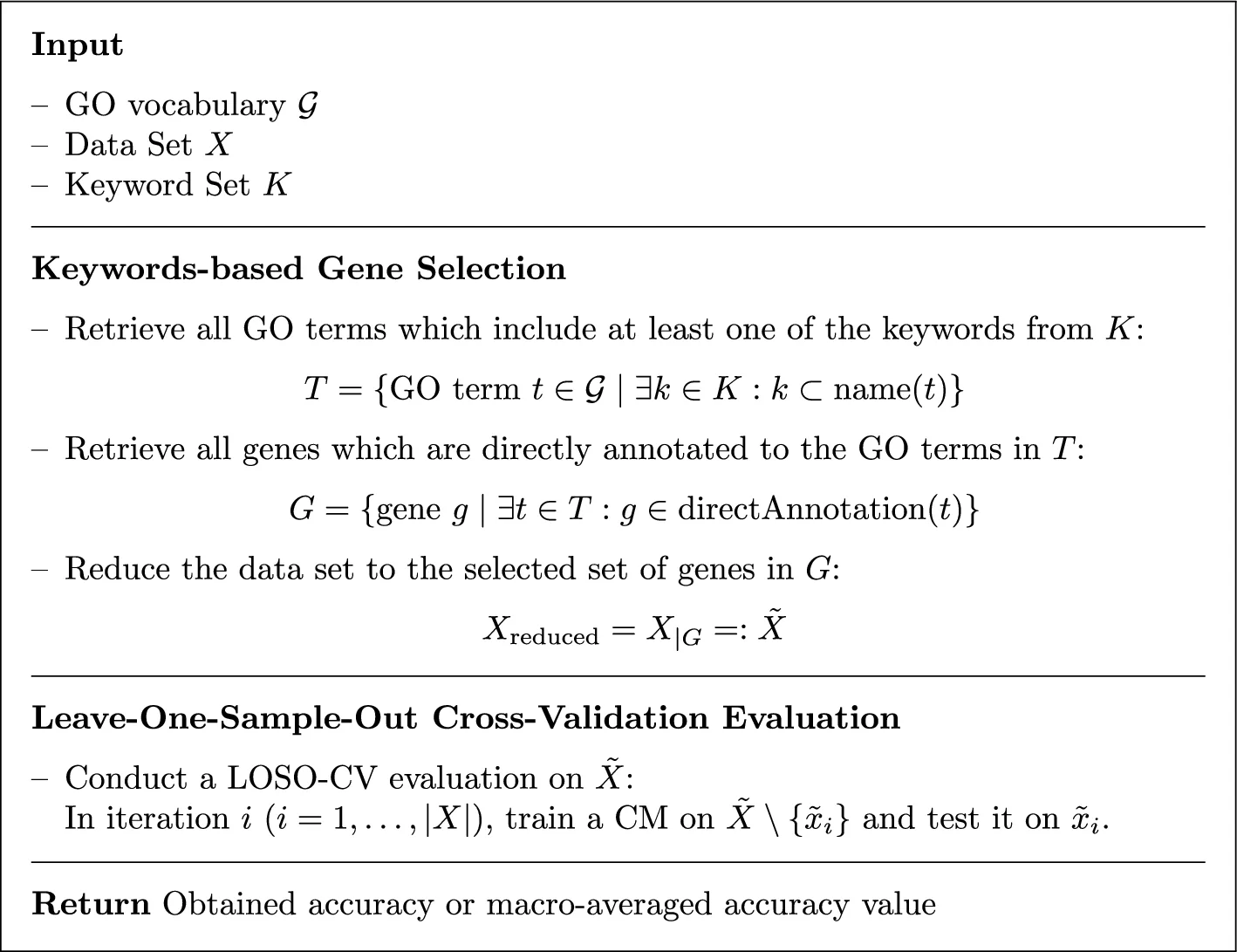
Evaluation Protocol. The keywords-based evaluation of each data set was following this evaluation procedure. CM: classification model

As our evaluation protocol, we applied the following experimental settings throughout this work, which are summarised at the end of the current section in Fig. 2.

*Classification Models*. For classification, we utilised Support Vector Machines (SVMs) with linear kernels [17], Random Forest (RF) ensembles with 100 unpruned decision trees [19], and Logistic Regression (LR) models with lasso (least absolute shrinkage and selection operator) penalty [38]. The weights of the Logistic Regression models were obtained through a training data-based cross-validation of 100 binary LR classifiers, with the maximum number of iterations set to $10^5$.

*Evaluation Approach*. For each data set, we applied the Leave-One-Sample-Out Cross-Validation (LOSO-CV) evaluation. The LOSO-CV evaluation consists of *n* training data/test data splits, whereby *n* denotes the number of data points in the corresponding data set. In each iteration of the evaluation, one data point is used as test data, while all the remaining data points are used to train the classification model.

*Performance Measures*. As discussed in “Methodology” section, we focused on the mean accuracy values for the balanced data sets, and on the mean macro-averaged accuracy values for the imbalanced data sets. Both measures are easy to interpret and provide a compact overview of the resulting outcomes.

*Baseline Comparisons*. We compared our proposed GO-based keywords-specific gene selection strategy against two baselines, the complete set of available genes and subsets of available genes obtained through random selection. To ensure a fair comparison with the random selection approach, we considered the following two points. First, for each data set, the number of randomly selected genes was equal to the number of genes corresponding to the best keywords-specific outcome. In case of a draw, i.e. two keyword sets leading to the best outcome, the number of randomly selected genes was set to the lower number of genes of both the best keywords-specific gene subsets. Second, to avoid under- or overestimating the random selection approach, we conducted 1,000 LOSO-CV evaluations and averaged the outcomes (one random selection of genes for one complete LOSO-CV evaluation).

*Implementation Tools*. As software, we used the programming language R (Version 4.3.3) with the non-basic packages (libraries) that are listed in the top part of Table 10 for the microarray data sets. For the RNA-Seq and DNAm data sets, we used Python (Version 3.12.6) with the non-basic packages that are listed in the bottom part of Table 10, in the appendix section “Appendix: Results for all keyword sets used”.

*Influenza Data Sets*. Note that for the Influenza A data sets, as described in “Appendix: Data set description” section, the gene expression data was recorded for different time stamps for each subject. In our experiments, we considered the data specific to each time stamp as one separate data set. More precisely, we conducted one LOSO-CV evaluation for each time stamp individually.

*Comments on Data Preprocessing*. Note that the GO Resource is updated on a regular basis, several times per year [39, 40]. With each release, new GO terms can be created, and existing GO terms merged or obsoleted. In this work, we are using fixed gene to GO term annotations which were obtained in November 2024 with the corresponding R package. For the use of Python, we manually downloaded an annotations file directly from the gene ontology website in January 2025.

*Instability of Random Forests and Variance of Cross-Validated LR Models*. Since RF and cross-validated LR classifiers are non-deterministic, we conducted 100 LOSO-CV evaluations for each data set-keyword combination. Therefore, the RF- and LR-specific results depicted in the subsequent section include the averaged performance values to ensure a fair comparison between the entire gene sets and our proposed keywords-based selection approach.

## Experiments: keywords-based gene selection

In the current section, we first list our choice of task-specific keyword sets for each data set, which is then followed by the presentation of the outcomes obtained.

### Choice of keywords (Microarray data sets)

Our experiments were conducted without any semantic input from experts of the classification tasks constituted by the corresponding data sets. We followed the three main steps of the gene selection approach that are described in detail in “Keywords-based gene selection” section. For the microarray data sets, we selected the following task-related keyword sets:

**Table:** 

**Pancreatic Cancer**:	$\{$’Pancreas’*\}*, $\{$’Pancreatic’*\}*, $\{$’Pancreas’, ’Pancreatic’*\}*;
**Prostate Cancer**:	$\{$’Testosterone’*\}*, $\{$’Estrogen’*\}*, $\{$’Prostate’*\}*;
**Alcoholism** (both):	$\{$’Alcohol’*\}*, $\{$’Liver’*\}*, $\{$’Liver’, ’Alcohol’*\}*;
**Influenza A** (both):	$\{$’Immune Complex’*\}*, $\{$’Immune System’*\}*, $\{$’Immune’*\}*.

For the choice of keywords, we would like to add the following comments. First, as an initial evaluation, we chose three keyword sets for each of the data sets. Second, for sets that consist of two keywords separated by a comma, i.e. $\{$‘Pancreas’, ‘Pancreatic’*\}* and $\{$‘Liver’, ‘Alcohol’*\}*, all GO terms are retrieved that contain at least one of both words in the GO term name. Third, the keyword set $\{$‘Immune’*\}* leads to a superset of the two keyword sets $\{$‘Immune Complex’*\}* and $\{$‘Immune System’*\}*, since each GO term name including ‘immune complex’ or ‘immune system’ obviously includes the word ‘immune’. Third, the search for GO terms was undertaken case insensitively.

### Choice of keywords for the ageing data set (Microarray)

For the Ageing data set, we initially planned to retrieve all GO terms containing the keywords ‘age’, ‘ageing’, or ‘aging’. However, this led to empty gene sets for the evaluation. Therefore, to overcome this issue, we focused on the characteristics of the available data set. As described in “Appendix: Data set description” section, the gene expression data was obtained from human brain tissues from frontal cortical regions. Thus, we opted for the keyword sets $\{$‘Cortical’*\}*, $\{$‘Cortex’*\}*, $\{$‘Forebrain’*\}*, and $\{$‘Brain’*\}*, analysing four instead of just three keyword sets. Note that, analogously to the keyword sets $\{$‘Immune’*\}*, $\{$‘Immune Complex’*\}* and $\{$‘Immune System’*\}*, the keyword set $\{$‘Brain’*\}* leads to a superset of the keyword set $\{$‘Forebrain’*\}*.

### Initial results (Microarray data)

The outcomes for the microarray data sets, i.e. Ageing, Pancreatic Cancer, Prostate Cancer, both Alcoholism data sets, as well as both Influenza A subtypes are depicted in Table 3, from which we can make the common main observation for the linear SVM and RF-based evaluations. First, each of the best keywords-specific gene subsets always outperformed the complete gene set while drastically reducing the initial feature dimension. For instance, in combination with the SVM model, for the Ageing, Pancreatic Cancer, and the Alcoholism data sets, the number of genes was reduced from 22,473 in total to 555, 124, as well as 123 (and 183), respectively. For the data sets Prostate Cancer and both Influenza A subtypes, the number of genes was reduced from 9,949 to 41, and from 14,196 to 120 (and 1,157), respectively. And second, for each of the seven separately analysed data sets, both the best keywords-specific and the entire gene set-specific outcomes always outperformed the random gene selection approach (see “Evaluation protocol” section). This shows that randomly reducing the feature dimension does not lead to an improvement in classification performance, not even in comparison to the very high-dimensional entire gene sets. For that reason, we did not repeat the evaluation of random gene selection in the upcoming evaluation of the RNA-Seq and DNAm data sets.

In combination with the Logistic Regression classifier, we observed the following differences. First, for the Pancreatic Cancer data set, none of our preselected three keyword sets led to an improvement over the entire gene set. Second, for the Influenza H1N1 data set, the random selection of 120 genes led to an improvement over the entire gene set. Third, since all of our three preselected keyword sets led to poor classification performances for the Alcoholism data set (w. ethanol treatment), we additionally included the rather generic keyword set $\{$‘Bolic’*\}*. This keyword set filters out metabolism-related GO terms, amongst others, that contain catabolic and metabolic processes, such as *carboxylic acid catabolic process* (GO:0046395) and *acyl-CoA metabolic process* (GO:0006637).

The results for the Alcoholism data set (w/o ethanol treatment) are omitted for the RF and LR models in Table 3 since both models failed to outperform the chance level with the chosen parameter settings (see “Evaluation protocol” section). We believe that this data set is rather noisy, as we can observe from the results obtained by SVM models. For this data set, the worst outcomes were obtained for all three categories, ‘Full’, ‘Best’, and ‘Rand’, whereby the gap to the second worst data set (Alcoholism w. ethanol treatment) is rather *large*, except for the random gene selection approach. Note that instead of conducting 100 evaluations in combination with the RF and LR models in the following experiments, for the RNA-Seq and DNAm data sets, we set the random seed to 42.

**Table 3:** Microarray Data – Accuracies in % (LOSO-CV evaluations). # G: Number of genes. # R: Reduced number of genes. Full: Results obtained in combination with the complete gene sets (# G genes). Best: Results obtained by the best keyword sets (# R genes). Rand: Results obtained by random selection of genes (# R genes – for Alc (w.) and H3N2, the smaller of the two # R values were taken). Influenza A: For the subtypes H3N2 and H1N1, the results are depicted for time stamps 101 h and 53 h, respectively (for these time stamps the best overall accuracy values were obtained). Bold values indicate the best-performing (highest accuracy) result in each row

Data Set	# G	Best Keyword Sets	# R	Full	Best	Rand
Ageing	22,473	$\{$’Brain’*\}*	555	87.8	**90**.**2**	82.9
Pancreas	22,473	$\{$’Pancreas’, ’Pancreatic’*\}*	124	81.9	**83**.**3**	77.8
Alc (w/o)	22,473	$\{$’Liver’*\}*	123	54.8	**61**.**9**	52.4
Alc (w.)	22,473	$\{$’Liver’*\}*, $\{$’Liver’, ’Alcohol’*\}*	123 / 183	61.9	**69**.**0**	54.8
H3N2	14,196	$\{$’Immune System’*\}*, $\{$’Immune’*\}*	120 / 1,157	88.2	**100**	82.4
H1N1	14,196	$\{$’Immune System’*\}*	120	78.3	**91**.**3**	69.6
Prostate	9,949	$\{$’Prostate’*\}*	41	66.4	**71**.**9**	60.9

Ageing	22,473	$\{$’Cortical’*\}*	142	87.8	**90**.**2**	81.0
Pancreas	22,473	$\{$’Pancreas’, ’Pancreatic’*\}*	124	**86**.**1**	82.6	81.7
Alc (w.)	22,473	$\{$’Bolic’*\}*	3,477	61.7	**65**.**0**	55.0
H3N2	14,196	$\{$’Immune System’*\}*, $\{$’Immune’*\}*	120 / 1,157	**100**	**100**	84.4
H1N1	14,196	$\{$’Immune System’*\}*	120	56.3	**65**.**4**	58.0
Prostate	9,949	$\{$’Prostate’*\}*	41	62.9	**72**.**4**	61.7

Ageing	22,473	$\{$’Cortex’*\}*	341	85.4	**86**.**7**	83.5
Pancreas	22,473	$\{$’Pancreas’, ’Pancreatic’*\}*	124	88.2	**88**.**9**	86.4
Alc (w.)	22,473	$\{$’Liver’*\}*	123	50.1	**57**.**5**	47.7
H3N2	14,196	$\{$’Immune’*\}*	1,157	96.2	**99**.**9**	93.7
H1N1	14,196	$\{$’Immune’*\}*	1,157	76.3	**78**.**9**	75.5
Prostate	9,949	$\{$’Estrogen’*\}*	149	67.2	**70**.**1**	66.8

Top: Linear SVMs. Middle: Logistic Regression. Bottom: Random Forests. For all accuracy (*A*) values obtained with SVMs and RFs, it holds: $A_{\text {Best}}> A_{\text {Full}} > A_{\text {Rand}}$

### Evaluation of RNA-Seq data sets

For the RNA-Seq data sets, we selected the following keyword sets, amongst others, including the generic keyword set $\{$‘RNA’*\}* for each of the data sets:

**Table:** 

**Liver Cancer**:	$\{$’Liver’*\}*, $\{$’Gland’*\}*, $\{$’Hepatocyte’*\}*, $\{$’Lipid’*\}*, $\{$’Blood’*\}*,
	$\{$’Gland’$\} \cap \{$’RNA’*\}*;
**Prostate Cancer**:	$\{$’Prostate’*\}*, $\{$’Testosterone’*\}*, $\{$’Estrogen’*\}*,
	$\{$’Estrogen’, ’Testosterone’*\}*, $\{$’Prostate’, ’RNA’*\}*,
	$\{$’Prostate’$\} \cap \{$’RNA’*\}*, $\{$’Testosterone’$\} \cap \{$’RNA’*\}*,
	$\{$’Estrogen’$\} \cap \{$’RNA’*\}*;
**Thyroid Cancer**:	$\{$’Thyroid’*\}*, $\{$’Epithelial’*\}*, $\{$’Thyroid’$\} \cap \{$’RNA’*\}*;
**Lung Cancer**:	$\{$’Immune’*\}*, $\{$’Astrocyte’*\}*, $\{$’Respiratory’*\}*, $\{$’Lung’*\}*,
	$\{$’Trachea’*\}*, $\{$’Astrocyte’, ’Diaphragm’*\}*,
	$\{$’Respiratory’, ’Lung’*\}*, $\{$’Respiratory’, ’Trachea’*\}*,
	$\{$’Trachea’$\} \cap \{$’RNA*\}*, $\{$’Respiratory’$\} \cap \{$’RNA’*\}*,
	$\{$’Lung’$\} \cap \{$’RNA’*\}*;
**Liver Disease**:	$\{$’Liver’*\}*, $\{$’Blood’*\}*, $\{$’Gland’*\}*, $\{$’Lipid’*\}*, $\{$’Hepatocyte’*\}*;
**Leukaemia**:	$\{$’Blood’*\}*, $\{$’Lymph’*\}*, $\{$’Plasma’*\}*.

The summarised outcomes for the RNA-Seq data sets are depicted in Table 4 (complete results are provided in the appendix, in Tables 11 and 12 for the SVM model, in Tables 15 and 16 for the LR model, as well as in Tables 19 and 20 for the RF model).

For the results obtained by linear SVM models, we can make the two main observations. First, similar to the evaluation of microarray data, the best keywords-specific gene subsets consistently outperformed the complete gene set while drastically reducing the initial feature dimension. More precisely, for the Liver, Prostate, Thyroid, and Lung Cancer data sets, the number of genes was reduced from 20,530 in total to 1,017, 4,847, 4,847, and 129, respectively. For the Liver Disease and Leukaemia data sets, the number of genes was reduced from 40,131 to 117 (768 and 5,334), and from 35,087 to 149, respectively. Second, the keyword set $\{$‘RNA’*\}* led to the best classification performance for two of the six data sets, Prostate and Thyroid Cancer, as well as for the Liver Disease data set, together with the keyword sets $\{$‘Liver’*\}* and $\{$‘Blood’*\}*. While the other applied keyword sets led to drastically reduced feature dimensions, ranging from 45 (with $\{$‘Prostate’*\}*) to 1,017 (with $\{$‘Lipid’*\}*) genes, the keyword set $\{$‘RNA’*\}* led to the largest gene subsets, including 4,847 genes for the data sets Prostate and Thyroid Cancer, and 5,334 genes for Liver Disease.

For the results obtained by Logistic Regression models, we can make the following three main observations. First, for the Liver Cancer data set, none of the keyword-specific gene subsets outperformed the complete gene set in terms of classification performance. However, the number of genes was substantially reduced from 20,530 to 358 and 712 for the keyword sets $\{$‘Gland’*\}* and $\{$‘Blood’*\}*, respectively, while achieving macro-averaged classification accuracies very close to that of the entire gene set, i.e. 99.6% vs 99.5%. Second, for the Liver Disease data set, both the complete gene set and the best-performing keyword-specific subsets achieved the same macro-averaged classification accuracy of 98.1%. At the same time, the number of genes was reduced from 40,131 to 364 and 1,134 for the keyword sets $\{$‘Gland’*\}* and $\{$‘Lipid’*\}*, respectively. Third, for all remaining data sets, the best keyword-specific gene subsets consistently outperformed the complete gene set while drastically reducing the initial feature dimension.

For the results obtained by Random Forests, we can make the following three main observations. First, for the Thyroid Cancer data set, none of the keyword-specific gene subsets outperformed the complete gene set. Nevertheless, the number of genes was reduced from 20,530 to 745 for the keyword set $\{$‘Epithelial’*\}*, with only a minor difference in macro-averaged accuracy of around 0.8% (94.7% vs 93.9%). Second, for the Leukaemia data set, both the complete gene set and the best-performing keyword-specific subsets achieved the same macro-averaged classification accuracy of 100%. Simultaneously, the number of genes was reduced from 35,087 to 149 and 772 for the keyword sets $\{$‘Lymph’*\}* and $\{$‘Blood’*\}*, respectively. Third, for all other data sets, the best keyword-specific gene subsets consistently outperformed the complete gene set while drastically reducing the initial feature dimension.

**Table 4:** RNA-Seq Data – Macro-averaged Accuracies in % (LOSO-CV evaluations). # G: Number of genes. # R: Reduced number of genes. Ca: Cancer, Ds: Disease. Full: Results obtained in combination with the complete gene sets (# G genes). Best: Results obtained by the best keyword sets (# R genes). Bold values indicate the best-performing (highest accuracy) result in each row

Data Set	# G	Best Keyword Sets	# R	Full	Best
Liver Ca	20,530	$\{$’Lipid’*\}*	1,017	99.5	**99**.**6**
Prostate	20,530	$\{$’RNA’*\}*	4,847	87.5	**89**.**4**
Thyroid	20,530	$\{$’RNA’*\}*	4,847	97.8	**97**.**9**
Lung	20,530	$\{$’Astrocyte’, ’Diaphragm’*\}*	129	99.4	**99**.**8**
Liver Ds	40,131	$\{$’Liver’*\}*, $\{$’Blood’*\}*, $\{$’RNA’*\}*	117 / 768 / 5,334	97.7	**97**.**9**
Leukaemia	35,087	$\{$’Lymph’*\}*	149	99.2	**100**

Liver Ca	20,530	$\{$’Gland’*\}*, $\{$’Blood’*\}*	358 / 712	**99**.**6**	99.5
Prostate	20,530	$\{$’RNA’, ’Prostate’*\}*	4,862	89.6	**90**.**9**
Thyroid	20,530	$\{$’RNA’*\}*	4,847	94.7	**98**.**0**
Lung	20,530	$\{$’Respiratory’, ’Lung’*\}*, $\{$’RNA’*\}*	388 / 4,847	98.9	**99**.**0**
Liver Ds	40,131	$\{$’Gland’*\}*, $\{$’Lipid’*\}*	364 / 1,134	**98**.**1**	**98**.**1**
Leukaemia	35,087	$\{$’Lymph’*\}*, $\{$’Blood’*\}*	149 / 772	99.2	**100**

Liver Ca	20,530	$\{$’Blood’*\}*	712	96.5	**97**.**6**
Prostate	20,530	$\{$’RNA’, ’Prostate’*\}*	4,862	78.1	**79**.**3**
Thyroid	20,530	$\{$’Epithelial’*\}*	745	**94**.**7**	93.9
Lung	20,530	$\{$’Respiratory’, ’Trachea’*\}*, $\{$’Immune’*\}*	240 / 1,220	98.5	**99**.**3**
Liver Ds	40,131	$\{$’Blood’*\}*	768	99.5	**99**.**8**
Leukaemia	35,087	$\{$’Lymph’*\}*, $\{$’Blood’*\}*	149 / 772	**100**	**100**

Top: Linear SVMs. Middle: Logistic Regression. Bottom: Random Forests

### Evaluation of DNAm data sets

For the DNAm data sets, we selected the following single-word keyword sets, amongst others, including the generic keyword sets $\{$‘DNA’*\}* and $\{$‘DNA Methylation’*\}* for each of the data sets (all evaluated keyword sets are provided in the appendix, including the corresponding outcomes obtained by linear SVM models in Tables 13 and 14, by LR models in Tables 17 and 18, as well as by RF models in Tables 21 and 22):

**Table:** 

**Tuberculosis**:	$\{$’Diaphragm’*\}*, $\{$’Immune’*\}*, $\{$’Bacteria’*\}*, $\{$’Granulom’*\}*,
	$\{$’Blood’*\}*, $\{$’Lung’*\}*, $\{$’Immune System’*\}*, $\{$’Tuberculosis’*\}*;
**Prostate Ca**:	$\{$’Prostate’*\}*, $\{$’Estrogen’*\}*, $\{$’Immune’*\}*, $\{$’Immune System’*\}*;
**Breast Ca**:	$\{$’Epithelial’*\}*, $\{$’Cellular’*\}*, $\{$’Hormone’*\}*, $\{$’Embryo’*\}*,
	$\{$’Estrogen’*\}*, $\{$’Immune’*\}*;
**HCC**:	$\{$’Protein’*\}*, $\{$’Cellular’*\}*, $\{$’Metabolic’*\}*, $\{$’Immune’*\}*,
	$\{$’Epithelial’*\}*, $\{$’Proliferation’*\}*, $\{$’Cytokine’*\}*.

The outcomes for the DNAm data sets are depicted in Table 5, from which we can make the following main observations for the SVM-based results.

First, similar to the microarray and RNA-seq data, the best keywords-specific gene subsets consistently outperformed the complete gene set while drastically reducing the initial feature dimension. Specifically, for the Tuberculosis, Prostate Cancer, Breast Cancer, and HCC data sets, the number of genes was reduced from 25,345 to 4, from 25,603 to 7, from 24,980 to 761, and from 23,980 to 13,520, respectively.

Second, the keyword sets $\{$‘DNA’*\}* and $\{$‘DNA Methylation’*\}* did not outperform the complete gene set, except for the Prostate Cancer data set, where the keyword $\{$‘DNA’*\}* yielded better performance while reducing the initial feature dimension from 25,603 to 3,749, although it was not the best-performing keyword overall.

Third, unlike for previous data types, for which unions of gene subsets derived from two keywords often outperformed the complete gene set, the intersections of keywords-specific gene subsets outperformed both the complete gene set and the individual keyword results for two data sets, Tuberculosis and Prostate Cancer. For the Tuberculosis data set, the best single keyword was $\{$‘Diaphragm’*\}*, reducing the initial feature dimension from 25,345 to 21 genes. However, the intersection of the gene subsets obtained using $\{$‘Diaphragm’*\}* and $\{$‘DNA’*\}* further reduced the initial feature dimension to only 4 genes (BASP1, TCF21, THBS1, WT1) and achieved superior performance compared to both the complete gene set and the individual keywords-specific sets. Similarly, for the Prostate Cancer data set, the best single keyword was $\{$‘DNA’*\}*, reducing the initial feature dimension from 25,603 to 3,749 genes. Yet, the intersection of the gene subsets associated with $\{$‘Immune’*\}* and $\{$‘Testosterone’*\}* reduced the feature dimension even further, to only 7 genes (GPI, IGF1R, NCF1, NMB, NQO1, SIRT1, THBS1), while outperforming both the complete gene set and the single-keyword subsets, even though neither of these keywords outperformed the complete gene set when evaluated individually.

Fourth, for the DNAm data sets, a larger number of keyword sets had to be tested before obtaining a subset that outperformed the complete gene set. This suggests that DNAm data require greater specificity in identifying relevant gene subsets and more targeted gene-selection strategies in order to achieve optimal classification performance.

For the results obtained by Logistic Regression models, we can make the same main observations as for the SVM models. The best keyword-specific gene subsets consistently outperformed the complete gene sets while drastically reducing the initial feature dimension. The same holds for the results obtained by Random Forests, except for the HCC and Breast Cancer data sets, for which none of the keyword-specific gene subsets outperformed the complete gene set. Nevertheless, the number of genes was reduced from 23,980 to 56 for the intersection of the gene subsets obtained using the keyword sets $\{$‘Cytokine’*\}*, $\{$‘Metabolic’*\}*, and $\{$‘Proliferation’*\}*, and from 24,980 to 182 for the gene subset obtained using the keyword $\{$‘Estrogen’*\}*, with only a minor difference in macro-averaged accuracy of 0.1% (66.3% vs 66.2%) and 0.2% (90.7% vs 90.5%) for the HCC and Breast Cancer data sets, respectively.

**Table 5:** DNAm Data – Standard & Macro-averaged Accuracies in % (LOSO-CV evaluations). **# G**: Number of genes. **# R**: Reduced number of genes. **Full**: Results obtained in combination with the complete gene sets (# G genes). **Best**: Results obtained by the best keyword sets (# R genes). **TB**: Tuberculosis. **Prost**: Prostate. Standard accuracy is used for the balanced data sets TB and Prost, for the imbalanced data sets Breast and HCC, macro-averaged accuracy values are depicted. Bold values indicate the best-performing (highest accuracy) result in each row

Data	# G	Best Keyword Sets	# R	Full	Best
TB	25,345	$\{$’Diaphragm’$\} \cap \{$’DNA’*\}*	4	80.0	**95**.**0**
Prost	25,603	$\{$’Immune’$\} \cap \{$’Testosterone’*\}*	7	60.4	**67**.**9**
Breast	24,980	$\{$’Epithelial’*\}*	761	92.7	**93**.**8**
HCC	23,980	$\{$’Protein’*\}*	13,520	70.3	**72**.**1**

TB	25,345	$\{$’Diaphragm’$\} \cap \{$’DNA’*\}*, $\{$’Lung’*\}*	4 / 186	65.0	**90**.**0**
Prost	25,603	$\{$’DNA’$\} \cap \{$’Immune’*\}*	304	62.3	**67**.**9**
Breast	24,980	$\{$’Epithelial’*\}*	761	90.9	**93**.**3**
HCC	23,980	$\{$’Cellular’, ’Epithelial’*\}*	6,927	65.2	**68**.**5**

TB	25,345	$\{$’Diaphragm’*\}*	21	60.0	**85**.**0**
Prost	25,603	$\{$’DNA’$\} \cap \{$’Immune’*\}*	304	50.9	**62**.**3**
Breast	24,980	$\{$’Estrogen’*\}*	182	**90**.**7**	90.5
HCC	23,980	$\{$’Cytokine’$\} \cap$ {’Metabolic’}$\cap \{$’Proliferation’*\}*	56	**66**.**3**	66.2

Top: Linear SVMs. Middle: Logistic Regression. Bottom: Random Forests

## Experiments: comparison against single GO terms

In the first part of the current section, we first compare the keywords-based gene selection approach to the selection of genes through individual GO terms. Subsequently, we discuss the potential of using individual GO terms to enhance existing gene expression knowledge.

### Keywords-based gene selection vs individual GO terms

In “Experiments: keywords-based gene selection” section, we demonstrated the proof of concept for our proposed keywords-based gene selection approach. In the current section, we want to analyse how well our method performed in comparison to the approach of selecting gene subsets based on individual GO terms. To this end, we conducted the following experiments.

We applied the same evaluation protocol as presented in “Evaluation protocol” Section. However, instead of retrieving GO terms which include at least one element from a preselected set of keywords in the GO term name, now, we focused on gene subsets that are directly annotated to a preselected individual GO term. More precisely, for each data set, we conducted the following evaluation steps, by iterating through all existing (i.e. more than 38,000) GO IDs:

i) Choose one individual GO term.
ii) Retrieve all genes directly annotated to the specific GO term.
iii) Conduct a LOSO-CV evaluation for the selected subset of genes.

Note that we only evaluated individual GO terms that led to a gene subset of at least two elements. Moreover, in this section, we focus on SVMs due to their deterministic nature.

#### Microarray data

To keep the experiments consistent with “Experiments: keywords-based gene selection” section, we chose the same time stamps for the Influenza A data sets, i.e. 101 h for H3N2 and 53 h for H1N1. The results for all microarray data sets and all individual GO terms are summarised in Table 6. From Table 6, we can make the following two main observations.

First, the keywords-based gene selection approach competes well with the gene selection based on individual GO terms (see column ‘Top (%)’). For the data sets Influenza A subtype H1N1 and Prostate Cancer, more than 99 % of individual GO terms were outperformed. For Ageing and Influenza A subtype H3N2, more than 98 % of individual GO terms were outperformed, while for the data set Alcoholism with ethanol treatment more than 96 % of individual GO terms were outperformed. For Pancreatic Cancer and Alcoholism without ethanol treatment, around 91 % and 86 % of the individual GO terms were outperformed, respectively.

Second, there exist individual GO terms that significantly outperform the keywords-based gene selection (see columns ‘Max’ and ‘# Max’). For instance, for the data sets Influenza A subtype H1N1 and Ageing, two GO terms led to an error-free LOSO-CV evaluation, respectively (all 23 H1N1 and all 41 Ageing samples were correctly classified).

**Table 6:** Microarray Data – Comparison Against All Individual GO Terms (with SVMs). Sam.: Number of samples in the data set. Corr.: Number of correctly classified samples with the keywords-based selection approach. Less/Equal/More: Number of individual GO terms leading to less/equal/more correct predictions (compared to the keywords-based approach). Max: Maximum amount of correct predictions obtained by individual GO terms. # Max: Number of GO terms leading to the maximal amount of correct predictions. Top (%): Implies that the keywords-based selection approach lies in the corresponding top % of individual GO term-specific outcomes

Data Set	Sam.	Corr.	Less	Equal	More	Max	# Max	Top (%)
H1N1 (53 h)	23	21	17,877	60	17	23	2	0.43
Prostate	128	92	16,578	46	77	98	6	0.74
– – – – – – – – – – – – – – – – – – – – – – – – – – – – – – – – – – – – – – – – – – – – – – – – – – – – – – – – – – – – –								
Ageing	41	37	18,548	137	141	41	2	1.48
H3N2 (101 h)	17	17	17,636	318	–	17	318	1.77
– – – – – – – – – – – – – – – – – – – – – – – – – – – – – – – – – – – – – – – – – – – – – – – – – – – – – – – – – – – – –								
Alc (w.)	42	29	18,147	287	392	38	2	3.61
– – – – – – – – – – – – – – – – – – – – – – – – – – – – – – – – – – – – – – – – – – – – – – – – – – – – – – – – – – – – –								
Pancreas	72	60	17,179	574	1,073	69	1	8.75
Alc (w/o)	42	26	16,190	891	1,745	36	2	14.0

The horizontal dashed lines were added to manually group the outcomes by column *Top (%)*

#### RNA-Seq data

The results for all RNA-Seq data sets and all individual GO terms are summarised in Table 7. From Table 7, we can make the following main observations.

As we already observed for the microarray data, the keywords-based gene selection approach competes well with the gene selection based on individual GO terms. For the data sets Thyroid, Liver, Lung, and Prostate Cancer, more than 99% of individual GO terms were outperformed. While for the Liver Disease data set, more than 89% of individual GO terms were outperformed. The Paediatric Leukaemia data set is a special case. At first glance, we seem to have only outperformed 81% of individual GO terms with the best performing of our keyword sets. However, this is due to the fact that, in addition to our best performing keyword set, 18.91% of individual GO terms led to a perfect LOSO-CV prediction.

**Table 7:** RNA-Seq Data – Comparison Against All Individual GO Terms (with SVMs). Ratio Neg.: Ratio of negative cases in the data set. MAA (%): Macro-averaged accuracy obtained with the keywords-based selection approach. Worse/Equal/Better: Number of individual GO terms leading to smaller/equal/greater MAA values (compared to the keywords-based approach). MAA Best: Maximum MAA value in %, obtained by individual GO terms. # Best Terms: Number of GO terms leading to the maximum MAA value. Top (%): Implies that the keywords-based selection approach lies in the corresponding top % of individual GO term-specific outcomes

Data Set	Ratio Neg.	MAA (%)	# GO Terms leading to			MAA Best	# Best Terms	Top (%)
			Worse	Equal	Better			
Lung	0.10	99.8	13,390	8	7	99.9	1	0.11
Liver Cancer	0.12	99.6	14,755	18	13	99.9	6	0.21
Thyroid	0.10	97.9	13,446	10	21	99.0	1	0.23
Prostate	0.09	89.4	13,420	7	50	93.2	1	0.42
– – – – – – – – – – – – – – – – – – – – – – – – – – – – – – – – – – – – – – – – – – – – – – – – – – – – – – – – – – – – –								
Liver Disease	0.11	97.9	12,236	225	1,149	100	82	10.10
– – – – – – – – – – – – – – – – – – – – – – – – – – – – – – – – – – – – – – – – – – – – – – – – – – – – – – – – – – – – –								
Leukaemia	0.11	100	11,001	2,565	–	100	2,565	18.91

The horizontal dashed lines were added to manually group the outcomes by column *Top (%)*

#### DNAm data

The results for all DNAm data sets and all individual GO terms are summarised in Table 8. From Table 8, we can make the following main observations.

As previously observed for the microarray and RNA-Seq data, the keywords-based gene selection approach competes well with the gene selection based on individual GO terms. For the data sets Tuberculosis and Breast Cancer, more than 99% of individual GO terms were outperformed. For the Prostate Cancer data set, more than 96% of individual GO terms were outperformed. Notably, for the HCC data set, all individual GO terms were outperformed, demonstrating that the keywords-based gene selection approach achieved an exceptionally strong classification result in that case. However, this result was obtained with the rather generic keyword ‘protein’, which is included in 2,416 GO terms, reducing the entire set of 23,980 genes to a subset of 13,520 genes in total.

**Table 8:** DNAm Data – Comparison Against All Individual GO Terms (with SVMs). Ratio Neg.: Ratio of negative cases in the data set. (MA)A (%): (Macro-averaged) accuracy obtained with the keywords-based selection approach. Worse/Equal/Better: Number of individual GO terms leading to smaller/equal/greater (MA)A values (compared to the keywords-based approach). (MA)A Best: Maximum (MA)A value in %, obtained by individual GO terms. # Best Terms: Number of GO terms leading to the maximum (MA)A value. Top (%): Implies that the keywords-based selection approach lies in the corresponding top % of individual GO term-specific outcomes. For data sets marked with an asterisk (*), the standard accuracy was used as performance measure

Data Set	Ratio Neg.	(MA)A (%)	# GO Terms leading to			(MA)A Best	# Best Terms	Top (%)
			Worse	Equal	Better			
HCC	0.54	72.1	13,516	–	–	71.1	1	0.00
– – – – – – – – – – – – – – – – – – – – – – – – – – – – – – – – – – – – – – – – – – – – – – – – – – – – – – – – – – – – –								
Breast	0.34	93.8	13,567	2	3	94.4	1	0.04
Tuberculosis*	0.50	95.0	13,531	17	3	100	3	0.15
– – – – – – – – – – – – – – – – – – – – – – – – – – – – – – – – – – – – – – – – – – – – – – – – – – – – – – – – – – – – –								
Prostate*	0.49	67.9	13,076	117	335	81.1	3	3.34

The horizontal dashed lines were added to manually group the outcomes by column *Top (%)*

### GO-based enhancement of gene expression knowledge

**Table 9:** Individual GO Terms Leading to the Maximum Amount of Correct Predictions (with SVMs). H1N1: Influenza A subtype H1N1 data set (53 h). Alcohol (w.)/(w/o): Alcoholism with/without ethanol treatment data sets. TB: Tuberculosis. # G: Number of genes in the current data set that are annotated to the corresponding GO term. GO ID: For reasons of legibility we omitted the prefix ’GO:’ in each row. H3N2/Liver Disease/Leukaemia: These data sets are omitted due to the high number of 318/82/2,565 best individual GO terms, respectively (see Tables 6 and 7). Top: Microarray data. Middle: RNA-Seq data. Bottom: DNAm data

Data Set	GO ID	GO Term Name	# G
H1N1	0097680	Double-strand break repair via classical non homologous and joining	8
	0001823	Mesonephros development	11
– – – – – – – – – – – – – – – – – – – – – – – – – – – – – – – – – – – – – – – – – – – – – – – – – – – – – – – – – – – – –			
Prostate	0061462	Protein localization to lysosome	6
	2001046	Positive regulation of integrin-mediated signaling pathway	10
	0007097	Nuclear migration	14
	1902176	NR$^{1}$ of oxidative stress-induced intrinsic apoptotic SP$^{2}$	15
	0031941	Filamentous actin	20
	0005875	Microtubule associated complex	28
– – – – – – – – – – – – – – – – – – – – – – – – – – – – – – – – – – – – – – – – – – – – – – – – – – – – – – – – – – – – –			
Ageing	0009792	Embryo development ending in birth or egg hatching	25
	0008201	Heparin binding	174
– – – – – – – – – – – – – – – – – – – – – – – – – – – – – – – – – – – – – – – – – – – – – – – – – – – – – – – – – – – – –			
Alcohol	0030742	GTP-dependent protein binding	20
(w.)	0045668	Negative regulation of osteoblast differentiation	49
– – – – – – – – – – – – – – – – – – – – – – – – – – – – – – – – – – – – – – – – – – – – – – – – – – – – – – – – – – – – –			
Pancreas	0045505	Dynein intermediate chain binding	36
– – – – – – – – – – – – – – – – – – – – – – – – – – – – – – – – – – – – – – – – – – – – – – – – – – – – – – – – – – – – –			
Alcohol	0051059	NF-kappaB binding	29
(w/o)	0010507	Negative regulation of autophagy	58

Thyroid	0005615	Extracellular space	1,444
– – – – – – – – – – – – – – – – – – – – – – – – – – – – – – – – – – – – – – – – – – – – – – – – – – – – – – – – – – – – –			
Liver	0001954	Positive regulation of cell-matrix adhesion	26
	0001657	Ureteric bud development	37
	0042594	Response to starvation	37
	0030855	Epithelial cell differentiation	94
	0007268	Chemical synaptic transmission	214
	0030424	Axon	318
– – – – – – – – – – – – – – – – – – – – – – – – – – – – – – – – – – – – – – – – – – – – – – – – – – – – – – – – – – – – –			
Prostate	0070507	Regulation of microtubule cytoskeleton organization	31
– – – – – – – – – – – – – – – – – – – – – – – – – – – – – – – – – – – – – – – – – – – – – – – – – – – – – – – – – – – – –			
Lung	0005975	Carbohydrate metabolic process	112

TB	0009308	Amine metabolic process	7
	0060539	Diaphragm development	9
	0061763	Multivesicular body-lysosome fusion	12
– – – – – – – – – – – – – – – – – – – – – – – – – – – – – – – – – – – – – – – – – – – – – – – – – – – – – – – – – – – – –			
Prostate	0010756	Positive regulation of plasminogen activation	7
	0004865	Protein serine/threonine phosphatase inhibitor activity	12
	0003676	Nucleic acid binding	109
– – – – – – – – – – – – – – – – – – – – – – – – – – – – – – – – – – – – – – – – – – – – – – – – – – – – – – – – – – – – –			
Breast	0046777	Protein autophosphorylation	129
– – – – – – – – – – – – – – – – – – – – – – – – – – – – – – – – – – – – – – – – – – – – – – – – – – – – – – – – – – – – –			
HCC	0005515	Protein binding	11,730

1: Negative regulation; 2: signaling pathway: Abbreviated by us due to reasons of space

**Fig. 3:**
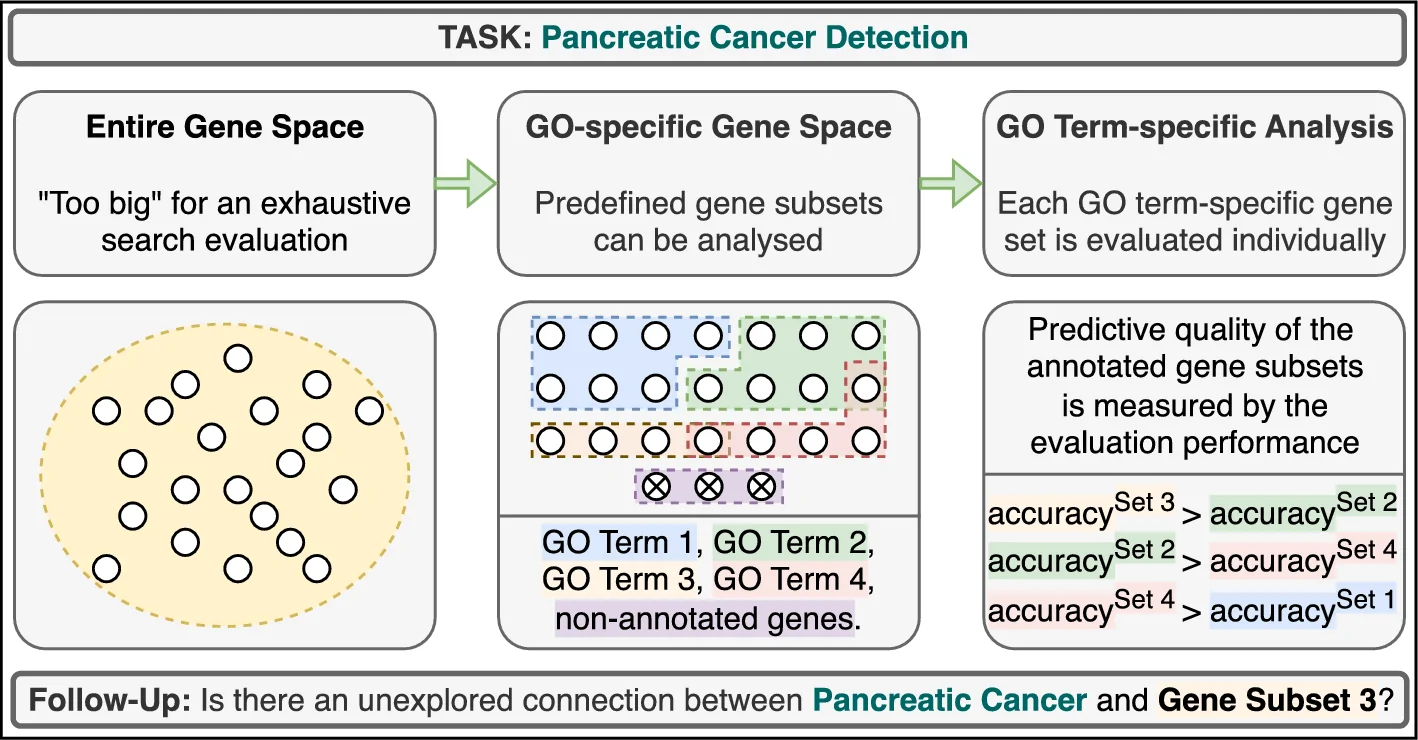
GO Term-based Mining for Connections between Diseases and Gene Sets

So far, we have used the GO structure to reduce the number of genes while simultaneously improving the classification performance of different tasks by choosing task-specific keywords. From Tables 6, 7, and 8, we were able to observe that there might be several subsets of genes which are able to outperform the semi-manually preselected gene subsets. While it is practically infeasible to evaluate all possible subsets from the available pool of genes, the Gene Ontology structure has the huge benefit that genes are already directly annotated to certain GO terms, and hence grouped semantically. This can be exploited to find potentially new connections between diseases and sets of genes, as illustrated in Fig. 3.

In Table 9, we state all GO terms that led to the best overall LOSO-CV evaluation results. Of course, the best outcomes most likely depend on a variety of factors and their synergy. More precisely, different choices of the kind of raw data preprocessing (type of normalisation and type of mean) and of the classification model (or even just different training parameters of the same model) might have led to varied outcomes and therefore to other best individual GO terms. An interesting observation in that direction can be made for the example task of prostate cancer detection, which we evaluated for all three data types, microarray, RNA-Seq, and DNAm. The number of best performing individual GO terms is six, one, and three, respectively. Moreover, not only the number of best performing individual GO terms is different, but the GO terms themselves all differ, including different genes. For instance, the best performing GO term for the RNA-Seq data, *regulation of microtubule cytoskeleton organization*, led to 31 genes, which are not included in any of the three best performing GO terms of the DNAm data. Therefore, the predictive efficacy of certain genes or gene groups is expected to also depend on the type of data. This observation might be especially interesting for future laboratory experiments, in which one can mine for novel well-predicting biomarkers, based on the type of omics data. Advanced molecular high-throughput techniques might shift the predictive importance of existing and already well-analysed genes.

Nevertheless, we analysed the identified best overall GO terms closer with respect to their biological meaning, as an example for the Ageing data set. For the Ageing data set, we found the two interesting GO terms *heparin binding* and *embryo development ending in birth or egg hatching*. The association between heparin and ageing is not surprising. Heparin levels increase with age [41, 42]. Although not a cause of ageing, the risk of developing Alzheimer’s disease (AD) increases with age, and an increase in heparin levels accelerates the formation of Amyloid beta fibres that are, among others, responsible for the development of AD [43]. Moreover, heparin-binding factors are potent neurotrophic factors that contribute to neuronal survival and alterations in their levels are associated with impaired memory or neurodegenerative disorders [44]. Instead, the GO term *embryo development ending in birth or egg hatching* sounds surprising at first glance. However, recent studies could show that age-dependent declines in genes and pathways associated with ageing also have a role in regulating embryonic development. Striking examples for this are HOX genes or genes associated with the Wnt signalling [45–47].

### Overlap analysis of best-performing individual GO terms

For the sake of completeness, for each data set with at least two best-performing individual GO terms (see Table 9), we took a closer look at the overlap of the corresponding gene sets.

In combination with the microarray data sets, we made the following observations. For the data sets H1N1 and Alcohol (w.), there was no overlap between the two best-performing GO terms, respectively. The two best-performing GO terms specific to the Ageing data set, *embryo development ending in birth or egg hatching* (25 genes) and *heparing binding* (174 genes) share the gene FGF2. Across the six best-performing individual GO terms of the Prostate Cancer data set, only *nuclear migration* (14 genes) and *microtubule associated complex* (28 genes) have an overlap of three genes, namely FBXW11, DCTN1, and PAFAH1B1. For the Alcohol (w/o) data set, the overlap of the two best-performing individual GO terms, *NF-kappaB binding* (29 genes) and *negative regulation of autophagy* (58 genes), consists solely of the gene EP300. Moreover, both of these GO terms overlap with one of the best-performing individual GO terms of the Alcohol (w.) data set, *negative regulation of osteoblast differentiation* (49 genes). More precisely, the intersections with *NF-kappaB binding* and *negative regulation of autophagy* lead to the single genes GSK3B and ERFE, respectively.

In combination with the RNA-Seq data sets, we made the following observations. Across the six best-performing GO terms of the Liver Cancer data set, gene intersections were observed only for a subset of GO-term pairs, while the remaining pairs showed no overlap. Notably, *axon* (318 genes) and *chemical synaptic transmission* (214 genes) exhibited the largest intersection, sharing 26 genes, including HTR2A, LPAR3, and SNCA. The GO terms *positive regulation of cell-matrix adhesion* (26 genes) and *ureteric bud development* (37 genes) share the gene SFRP1. The GO terms *positive regulation of cell-matrix adhesion* (26 genes) and *axon* (318 genes) share the genes CIB1 and GSK3B. The GO terms *ureteric bud development* (37 genes) and *epithelial cell differentiation* (94 genes) share the genes FGFR2, SIX1, TCF21, and WT1. The GO terms *ureteric bud development* (37 genes) and *axon* (318 genes) share the genes RET and TGFB1. The GO terms *response to starvation* (37 genes) and *chemical synaptic transmission* (214 genes) share the gene HCRT. The GO terms *response to starvation* (37 genes) and *axon* (318 genes) share the genes ACADM and ULK1. The GO terms *epithelial cell differentiation* (94 genes) and *axon* (318 genes) share the genes ACTL8 and TGFB2.

**Fig. 4:**
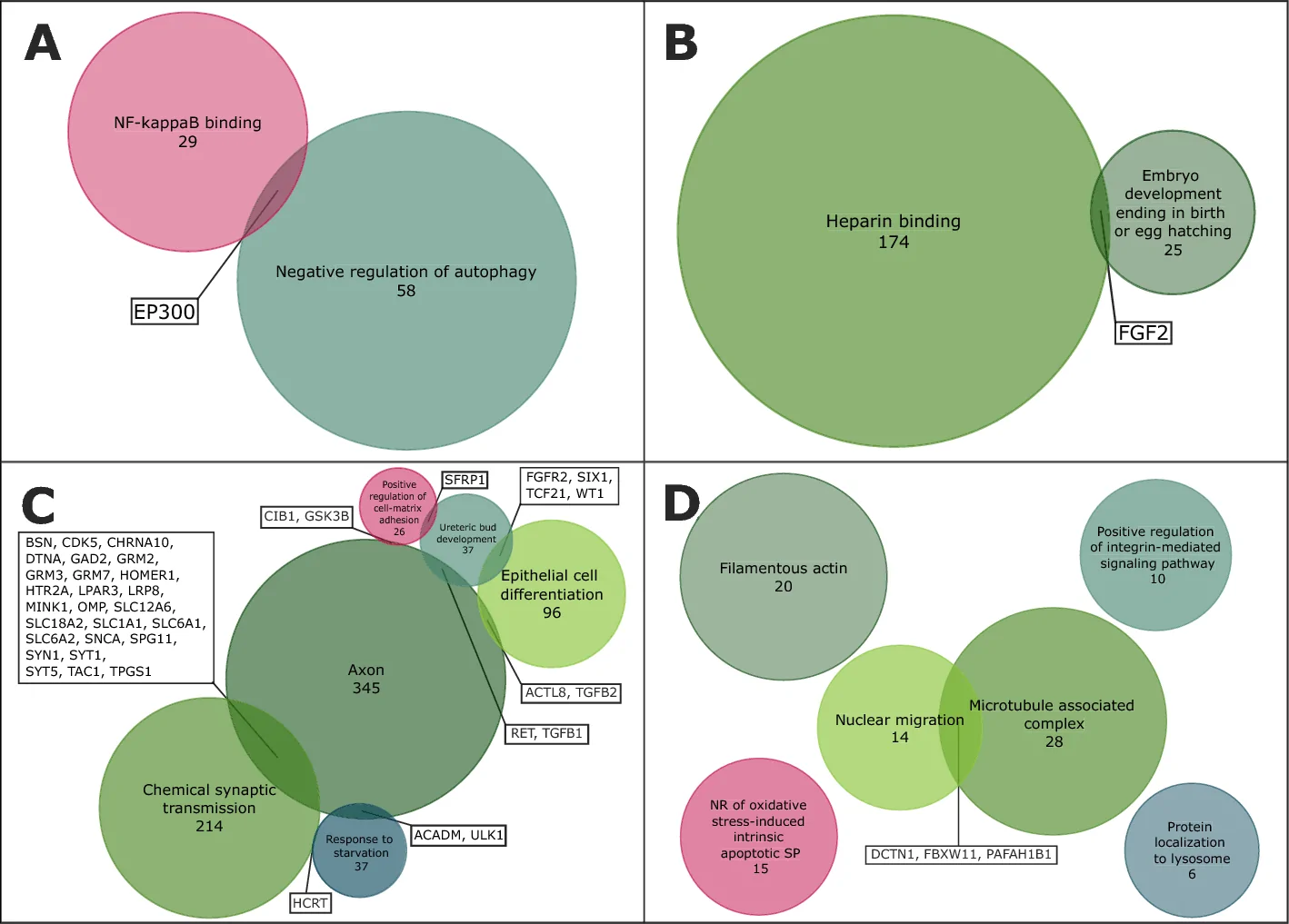
Overlaps of gene sets between all best-performing GO terms, which are reported in Table 9. **A**: Alcoholism (without ethanol treatment). **B**: Ageing. **C**: Liver Cancer. **D**: Prostate Cancer (miArr)

For the DNAm data sets, the three best-performing GO terms exhibited no overlap in neither the Tuberculosis nor Prostate Cancer data sets. All overlaps are summarised as area-proportional Euler diagrams [48] in Fig. 4, which was obtained using the VennMaster tool [49].

## Discussion

Previous research has explored various strategies for incorporating Gene Ontology information into computational models for disease gene analysis and classification. In the study conducted by Asif et al. [16], a machine learning framework was proposed to identify disease-related genes associated with human disorders. The framework quantifies functional similarities among genes by computing Gene Ontology semantic similarity scores using established measures such as Wang, Resnik, and Relevance. The prediction task was formulated as a binary classification problem, where known disease-associated genes were treated as positive instances and non-disease genes were considered negative instances. To support this classification, pairwise GO similarity matrices were constructed and used as input features for training supervised learning models, including Support Vector Machines and Random Forests, amongst others. The Random Forest classifier achieved the best performance. Furthermore, the framework was able to identify novel candidate genes enriched for disease-related phenotypes. Overall, this study demonstrated that GO-based semantic similarity provides a biologically meaningful feature representation that can improve disease gene prediction while reducing the dimensionality of raw molecular data.

Pritykin et al. [20] developed a systematic computational framework to identify multifunctional genes across the genomes of humans, flies, and yeast using Gene Ontology Biological Process annotations. Unlike conventional approaches that rely on simple counts of GO terms per gene, their method focuses on identifying genes associated with multiple functionally distinct biological processes. To achieve this, the authors applied statistical measures to quantify the functional dissimilarity between GO terms, incorporating GO hierarchy propagation and orthology information to ensure robust cross-species comparisons. This strategy enabled the identification of genes linked to at least two distinct biological processes, representing approximately 15–25% of the genes analysed. The study further demonstrated that multifunctional genes exhibit distinctive biological and molecular properties. These genes are more likely to encode intrinsically disordered proteins, show broader expression across tissues, display higher evolutionary conservation, and occupy central positions within protein–protein interaction networks. Moreover, multifunctional genes were found to be significantly enriched among genes associated with human diseases, including those documented in the Online Mendelian Inheritance in Man (OMIM) database. These findings underscore the value of Gene Ontology annotations for revealing functional complexity and highlighting the biological importance of genes involved in multiple distinct processes.

While the most related prior works, including the studies discussed above, have primarily used GO for gene functional similarity [16] or multifunctionality characterisation [20], our study offers two distinct approaches. First, we introduced a keywords-based gene selection approach in which we filter out GO terms that contain at least one word from a preselected set of keywords in the GO term name. We evaluated our proposed method on sixteen publicly available benchmark data sets from the Gene Expression Omnibus (GEO) and Kaggle in combination with linear Support Vector Machines (SVMs), Random Forests (RFs), and Logistic Regression (LR) models by conducting Leave-One-Sample-Out Cross-Validation (LOSO-CV) evaluations. The obtained outcomes indicate that the size of the initial feature space (number of gene expression values) can be drastically reduced, while simultaneously improving classification performance, in general. The main advantage is that the lack of expert knowledge for selecting well-performing gene subsets is overcome by the task-specific expert knowledge which is already implicitly provided through the GO structure. Furthermore, we could observe that it might be even sufficient to preselect straightforward task-specific keywords to improve classification accuracy, such as by choosing the keywords ‘liver’ and ‘prostate’ for the detection of alcoholism and prostate cancer, respectively. Based on the evaluation of the Ageing data set, we provided another option for preselecting beneficial keywords, i.e. by focusing on the type of data, e.g. tissue samples from specific organs. Another advantage is that no statistics have to be computed for the ranking of features, which might be especially cumbersome if the number of samples is scarce. Moreover, the semantics-based selection approach is independent of any classification model, which drastically lowers the risk of overfitting, while the Gene Ontology knowledge always keeps the choice of features explainable by definition. However, the main disadvantage is that poorly chosen keywords might lead to a deterioration of classification performance, as can be observed in the appendix, in Tables 11–22.

Second, while it is not feasible in practice to evaluate all possible gene combinations to find task-important gene subsets due to the huge amount of annotated genes, it is possible to analyse the whole GO space by iterating through all GO terms separately. As we already discussed in the second experimental part of the current manuscript (in “Experiments: comparison against single GO terms” Section), the performance of gene subsets most likely depends on several factors and their synergy (choice of data preprocessing steps, classification model and model parameters, evaluation protocol, etc.), including the attributes of the gene expression data and task itself, e.g. the distribution of class labels and meta data characteristics, such as age or sex. Nevertheless, genes annotated to well-performing GO terms could be further analysed in laboratory experiments, leading to new insights on biological mechanisms.

Note that our proposed GO term-based gene selection approach can also be used as a pre-filtering method for or by task-specific experts. As a first step, the experts could choose initial sets of detailed keywords, which would lead to a specific subset of genes. Subsequently, the obtained reduced gene subset can be further manually partitioned by the expert users, or even extended by genes that were left out by the corresponding combination of keywords.

Inspired by the effectiveness of individual GO terms, one could also combine the keywords-based gene selection and a restricted GO term space exploration as an alternative to the GO term-based exhaustive search. To this end, one could select several sets of keywords and iterate only through all individual GO terms that include at least one of the preselected keywords.

## Conclusion

In this work, we utilised the Gene Ontology (GO) knowledge base in two distinct scenarios for gene selection by making use of the fact that the genes in the GO vocabulary are structured into semantically meaningful subgroups, called GO terms, based on their functions and characteristics. We showed that the GO knowledge base can be exploited in combination with different types of data and programming languages. To this end, we utilised R for the analysis of the microarray data sets, and Python for the evaluation of the RNA-Seq and DNAm data, as described in “Evaluation protocol” section. It is worth mentioning that we repeated the experiments with both programming languages, observing no difference in performance between them. Utilising the GO ontology, drastically decreased the initial high-dimensional feature spaces while maintaining classification performance, and, in most cases, even outperforming the entirely available gene sets.

Future work will focus on optimising keyword selection rather than relying on manual methods, expanding the analysis to the validation of the biological relevance of the findings. For instance, as for the Ageing data set, one could start from the data set description and use a Natural Language Processing (NLP) approach for the choice of keywords, e.g. a Large Language Model (LLM) could generate sets of keywords [50], which are then parsed into the system. In addition to Gene Ontology terms, we also plan to incorporate additional biological knowledge bases, including KEGG (Kyoto Encyclopedia of Genes and Genomes) [51] and Reactome [52] pathways, to enhance the gene filtering process and improve the identification of genes relevant to the target disease. Further options could be the identification of potential risk genes for specific diseases with deep learning approaches such as by Lu et al. [53] that use PPI networks and mutation data in conjunction with deep reinforcement learning for clear cell renal cell carcinoma. Here, the uncertainty in the PPI network could be stabilised [54] and augmented by additional semantic information.

## Data Availability

Availability of data and materials. All data sets utilised in this article are publicly available from the Gene Expression Omnibus (GEO) as well as from Kaggle. The GEO data sets are available under the accession IDs and links stated below: GSE53890, The Ageing miArr data set: https://www.ncbi.nlm.nih.gov/geo/query/acc.cgi?acc=GSE53890. GSE15471, The Pancreatic Carcinoma miArr data set: https://www.ncbi.nlm.nih.gov/geo/query/acc.cgi?acc=GSE15471. GSE52553, The Alcoholism miArr data set: https://www.ncbi.nlm.nih.gov/geo/query/acc.cgi?acc=GSE52553. GSE6606 and GSE6608, The Prostate Carcinoma miArr data set: https://www.ncbi.nlm.nih.gov/geo/query/acc.cgi?acc=GSE6606, https://www.ncbi.nlm.nih.gov/geo/query/acc.cgi?acc=GSE6608. GSE52428, The Influenza miArr data set: https://www.ncbi.nlm.nih.gov/geo/query/acc.cgi?acc=GSE52428. GSE263786, The Liver Disease RNA-Seq data set: https://www.ncbi.nlm.nih.gov/geo/query/acc.cgi?acc=GSE263786. GSE227832, The Paediatric Leukaemia RNA-Seq data set: https://www.ncbi.nlm.nih.gov/geo/query/acc.cgi?acc=GSE227832. GSE304107, The Tuberculosis DNAm data set: https://www.ncbi.nlm.nih.gov/geo/query/acc.cgi?acc=GSE304107. GSE262524, The Prostate Carcinoma DNAm data set: https://www.ncbi.nlm.nih.gov/geo/query/acc.cgi?acc=GSE262524. GSE225845, The Breast Carcinoma DNAm data set: https://www.ncbi.nlm.nih.gov/geo/query/acc.cgi?acc=GSE225845. GSE281691, The Hepatocellular Carcinoma (HCC) DNAm data set: https://www.ncbi.nlm.nih.gov/geo/query/acc.cgi?acc=GSE281691. The Liver, Prostate, Thyroid, and Lung Carcinoma RNA-Seq data sets are available from Kaggle: https://www.kaggle.com/datasets/tianjiechen/tcga-rna-datasets. In addition to the data, the code is publicly available on Zenodo: https://zenodo.org/records/19087304.

## Appendix: Data set description

*The Ageing miArr data set (GSE53890)* is a gene expression profiling study that was performed using Affymetrix Human Genome HG U133 Plus 2.0 microarrays. It consists of post-mortem human brain tissue samples from the cortical regions of 41 healthy adults. Tissue samples were collected in the Rush University Medical Center, the University of Maryland, Duke University, the Brigham and Women’s Hospital, and the Massachusetts General Hospital. Brain samples with a tissue pH < 6.5 or post-mortem interval >20 h were excluded. The sex of the individuals was balanced (21 females, 20 males) and the subjects’ age ranged from 24 to 106 years. The authors [26] arranged the individuals into four clusters according to their age, including 12 young (< 40 years, five males, seven females), nine middle aged (40–69 years, five males, four females), 16 normal aged (70–94 years, nine males, seven females), and four extremely aged (95–106 years, one male, three females). We separated the subjects into two equally distributed age groups, below 69 years (21 subjects, ten males, eleven females) and above 69 years (20 subjects, ten males, ten females).

*The Pancreatic Carcinoma miArr data set (GSE15471)* provides gene expression analyses performed on an Affymetrix HG U133 Plus 2.0 whole genome microarray. It consists of 36 paired human samples of tumour and normal pancreas tissue as well as three replicates of these pairs. Tissue samples were extracted at the time of surgery from resected pancreas at the Clinical Institute Fundeni [27]. As we performed leave-one-out cross-validation, and thus need to have independent training and test sets, we only considered the replicates of the paired samples.

*The Alcoholism miArr data set (GSE52553)* is based on immortalised lymphoblastoid cell lines (LCLs), which were created from the peripheral blood mononuclear cells of alcoholic and non-alcoholic individuals. Alcoholics were defined as individuals who met the criteria for alcohol dependence as defined in the DSM-IV, while non-alcoholics were defined as individuals who had consumed at least one alcoholic drink and did not meet any of the following four definitions for alcohol dependence [28]: DSM-IV [55], DSM-IIIR [56], ICD-10 [57], or Feighner’s criteria for definite alcoholism [58]. There were twelve males and nine females in each group (alcoholic and non-alcoholic). Each cell line was cultured for 24 h, both with and without 75 mM ethanol, and gene expression was measured using an Affymetrix HG U133 Plus 2.0 microarray. Here, we split the data set into two parts (with and without ethanol treatment) and analysed the data sets individually.

*The Prostate Carcinoma miArr data set (GSE6606 & GSE6608)* derives from the SuperSeries GSE6919, which was performed using multiple types of microarray. For our analysis, we focused on gene expression profiles obtained using the Affymetrix Human Genome U95 Version 2 microarray and the subseries GSE6606, which comprises 65 primary prostate tumour samples, and GSE6608, which contains 63 samples of normal prostate tissue adjacent to the tumours [29].

*The Influenza miArr data set (GSE52428)* comprises healthy volunteers who were inoculated with either influenza A H1N1 or H3N2. Peripheral blood was extracted at baseline and every eight hours for seven days. Affymetrix Human Genome U133A 2.0 microarrays were used to measure the gene expression of the peripheral blood cells [30]. 24 and 17 subjects participated in the data collection for the Influenza A subtypes H1N1 and H3N2, respectively. Note that four time points of the gene expression profile of one participant are missing for the H1N1 subtype. Furthermore, for the H3N2 subtype, one sample lacks three measurements and two samples lack one measurement. The volunteers can be split into two groups: those who developed symptoms and those who did not. Both groups are distributed equally according to the influenza A subtypes H1N1 and H2N2. For our analysis, we assessed the H1N1 and H3N2 subtypes of influenza A separately.

*The Liver, Prostate, Thyroid, and Lung Carcinoma RNA-Seq data sets (Kaggle)* have recently been analysed in [31] by Chen and Kabir. The authors made the $\log _{2}(x+1)$-normalised Illumina HiSeq (not further specified) RNA-sequencing data publicly available on Kaggle under the link that is provided in Table 2. Tissues with primary, recurrent, or metastatic tumour were combined to one class, called *Tumour*. Samples lacking RNA-sequencing information were excluded, and all data sets underwent min-max normalisation prior to analysis.

*The Liver Disease RNA-Seq data set (GSE263786)* comprises RNA-sequencing data from normal and diseased liver tissue samples taken from multiple liver regions [32]. Gene expression was measured using an Illumina NovaSeq 6000 instrument.

*The Paediatric Leukaemia RNA-Seq data set (GSE227832)* is an RNA-sequencing data set comprising bone marrow aspirates or peripheral blood samples collected from patients with primary paediatric acute lymphoblastic leukaemia (ALL). The data set contains samples taken at diagnosis, relapse and remission, as well as from healthy controls. Depending on the RIN value and the available input RNA, the authors [33] used three different sequencing technologies: Illumina HiSeq 2000, Illumina MiSeq, and Illumina HiSeq 2500. To define a clear binary classification task, we restricted our analyses to ALL samples at diagnosis and healthy donor samples measured with Illumina HiSeq2500. Furthermore, we disregarded one sample and included its technical replicate due to applying a leave-one-out cross-validation evaluation, and excluded the samples labelled as ‘validation’.

*The Tuberculosis DNAm data set (GSE304107)* comprises 30 plasma samples from people living with HIV [34]. Each of the subjects had active, latent, or no tuberculosis. In our analysis, we focused on the binary task *active* vs *no* tuberculosis, including ten samples each. The data were collected using the Illumina Infinium MethylationEPIC Version 2 device.

*The Prostate Carcinoma DNAm data set (GSE262524)* is composed of prostate tumour samples and adjacent tissue samples [35]. In addition to paired-end sequencing using an Illumina HiSeq 2500 device (GSE237995), the data set collectors performed DNA methylation assays using two arrays that covered different numbers of methylation sites. They analysed the DNA methylation of five tumour and adjacent tissue samples from European Americans, as well as 30 non-tumour and 32 tumour samples from African Americans, using the Illumina Infinium Human Methylation450 BeadChip DNA methylation array (GSE262522). This array covers approximately 450,000 methylation sites. However, in our analysis, we use the data additionally obtained by the Illumina Infinium MethylationEPIC array, covering *∼ *850,000 methylation sites, to measure the DNA methylation profile of 27 tumour samples and 26 adjacent tissue samples from European Americans (GSE262524).

*The Breast Carcinoma DNAm data set (GSE225845)* is a collection of samples containing normal, tumour, as well as adjacent to normal breast tissues [36]. We focused on normal and tumour cells, including 113 and 224 samples, respectively. The data were obtained using the Illumina Infinium MethylationEPIC array.

*The Hepatocellular Carcinoma (HCC) DNAm data set (GSE281691)* comprises blood leukocyte DNA samples from metabolic HCC cases and from HCC controls with metabolic liver disease, collected by the Illumina Infinium MethylationEPIC device [37]. The number of HCC cases and HCC controls is equal to 221 and 260, respectively.

## Appendix: Results for all keyword sets used

Table 10 contains all R and Python packages used in our implementation. In Tables 11 and 12 (SVM models), 15 and 16 (LR models), as well as 19 and 20 (RF models), we state the obtained MAA values for all RNA-Seq data sets and for all keyword sets used in our experiments. Tables 13 and 14 (SVM models), 17 and 18 (LR models), as well as 21 and 22 (RF models) include the results for all keyword sets used in combination with the DNAm data sets.

**Table 10:** Libraries and functions used within the Prog. Languages R (top) and Python (bottom)

Package	Functions	Used For
biomaRt	useMart	Loading the GO knowledge base (desired organism)
[59, 60]	getBM	Retrieving GeneChip & gene to GO term mappings
– – – – – – – – – – – – – – – – – – – – – – – – – – – – – – – – – – – – – – – – – – – – – – – – – – – – – – – – – – – – –		
	read.affybatch	Reading and loading CEL files
affy [61]	mas5	Applying MAS5 normalisation on raw CEL files
	exprs	Computing expression values from cell intensities
– – – – – – – – – – – – – – – – – – – – – – – – – – – – – – – – – – – – – – – – – – – – – – – – – – – – – – – – – – – – –		
e1071 [62]	svm	Designing support vector machine models
randomForest [63]	randomForest	Designing random forest models
glmnet [64, 65]	cv.glmnet	Designing cross-validated logistic regression models
caret [66]	confusionMatrix	Computing confusion matrices

mygene$^{+}$ [67–69]	querymany	Mapping gene Ensembl IDs to gene names
goatools [70]	obo_parser	Parsing GO files to access term relationships
– – – – – – – – – – – – – – – – – – – – – – – – – – – – – – – – – – – – – – – – – – – – – – – – – – – – – – – – – – – – –		
sklearn [71]	LeaveOneOut	Performing cross-validation leaving one sample out
	StandardScaler	Normalising features before passing them to SVMs
	SVM	Designing support vector machine models
	RandomForestClassifier	Designing random forest classification models
	LogisticRegressionCV	Designing cross-validated logistic regression models
	confusion_matrix	Computing confusion matrices

$^{+}$ URL: https://mygene.info/ (Last accessed on March 9, 2026)

**Table 11:** RNA-Seq Data – Macro-averaged Accuracies in % (LOSO-CV with lin. **SVM Models**). *\emptyset *: Entire gene set. Keyword sets for which the MAA values were at least as high as obtained with the entire gene set are marked with circles (*∘ *). The best overall results are highlighted in bold

Keyword Set	# Genes	TN	FP	FN	TP	MAA (%)
*Thyroid Cancer Data Set*						
*\emptyset *	20,530	57	2	5	508	97.8
$\{$**’RNA’***\}*	**4,847**	**57**	**2**	**4**	**509**	**97**.**9**
$\{$’Epithelial’*\}*	745	57	2	7	506	97.6
$\{$’Thyroid’*\}*	133	55	4	11	502	95.5
$\{$’Thyroid’$\} \cap \{$’RNA*\}*	76	52	7	16	497	92.5

*Liver Cancer Data Set*						
*\emptyset *	20,530	50	0	4	368	99.5
$\{$**’Lipid’***\}*	**1,017**	**50**	**0**	**3**	**369**	**99**.**6**
$\{$’Blood’$\}^{\circ }$	712	50	0	4	368	99.5
$\{$’RNA’$\}^{\circ }$	4,847	50	0	4	368	99.5
$\{$’Gland’*\}*	358	49	1	4	368	98.5
$\{$’Liver’*\}*	117	47	3	2	370	96.7
$\{$’Hepatocyte’*\}*	130	47	3	5	367	96.3
$\{$’Gland’$\} \cap \{$’RNA*\}*	177	46	4	6	366	95.2

*Prostate Cancer Data Set*						
*\emptyset *	20,530	40	12	10	488	87.5
$\{$**’RNA’***\}*	**4,847**	**42**	**10**	**10**	**488**	**89**.**4**
$\{$’Estrogen’*\}*	174	36	16	14	484	83.2
$\{$’Testosterone’*\}*	73	37	15	24	474	83.2
$\{$’Prostate’*\}*	45	34	18	15	483	81.2
$\{$**’Prostate’, ’RNA’***\}*	**4,862**	**42**	**10**	**10**	**488**	**89**.**4**
$\{$’Estrogen’, ’Testosterone’*\}*	226	37	15	12	486	84.4
$\{$’Prostate’$\} \cap \{$’RNA*\}*	30	33	19	12	486	80.5
$\{$’Estrogen’$\} \cap \{$’RNA*\}*	81	34	18	23	475	80.4
$\{$’Testosterone’$\} \cap \{$’RNA*\}*	26	30	22	12	486	77.6

*Lung Cancer Data Set*						
*\emptyset *	20,530	109	1	2	987	99.4
$\{$’Astrocyte’$\}^{\circ }$	108	110	0	6	983	99.7
$\{$’Immune’$\}^{\circ }$	1,220	109	1	2	987	99.4
$\{$’Respiratory’*\}*	223	107	3	7	982	98.3
$\{$’RNA’*\}*	4,847	106	4	4	985	98.0
$\{$’Lung’*\}*	182	105	5	4	985	97.5
$\{$’Trachea’*\}*	19	102	8	12	977	95.8
$\{$**’Astrocyte’, ’Diaphragm’***\}*	**129**	**110**	**0**	**4**	**985**	**99**.**8**
$\{$’Respiratory’, ’Lung’*\}*	388	107	3	4	985	98.4
$\{$’Respiratory’, ’Trachea’*\}*	240	107	3	5	984	98.4
$\{$’Respiratory’$\} \cap \{$’RNA*\}*	42	109	1	6	983	99.2
$\{$’Lung’$\} \cap \{$’RNA*\}*	100	107	3	4	985	98.4
$\{$’Trachea’$\} \cap \{$’RNA*\}*	13	105	5	8	981	97.3

This table depicts the results for the four Kaggle data sets

**Table 12:** RNA-Seq Data – Macro-averaged Accuracies in % (LOSO-CV with lin. **SVM Models**). *\emptyset *: Entire gene set. Keyword sets for which the MAA values were at least as high as obtained with the entire gene set are marked with circles (*∘ *). The best overall results are highlighted in bold

Keyword Set	# Genes	TN	FP	FN	TP	MAA (%)
*Leukaemia Data Set*						
*\emptyset *	35,087	8	0	1	64	99.2
$\{$**’Lymph’***\}*	**149**	**8**	**0**	**0**	**65**	**100**
$\{$’Blood’$\}^{\circ }$	772	8	0	1	64	99.2
$\{$’Plasma’$\}^{\circ }$	5,685	8	0	1	64	99.2
$\{$’RNA’$\}^{\circ }$	5,358	8	0	1	64	99.2

*Liver Disease Data Set*						
*\emptyset *	40,131	26	1	2	214	97.7
$\{$**’Liver’***\}*	**117**	**26**	**1**	**1**	**215**	**97**.**9**
$\{$**’Blood’***\}*	**768**	**26**	**1**	**1**	**215**	**97**.**9**
$\{$**’RNA’***\}*	**5,334**	**26**	**1**	**1**	**215**	**97**.**9**
$\{$’Lipid’$\}^{\circ }$	1,134	26	1	2	214	97.7
$\{$’Gland’*\}*	364	26	1	3	213	97.5
$\{$’Hepatocyte’*\}*	130	26	1	3	213	97.5

This table depicts the results for the two GEO data sets

**Table 13:** DNAm Data – Accuracies (Acc) in % (LOSO-CV with lin. **SVM Models**). *\emptyset *: Entire gene set. Keyword sets for which the Acc values were at least as high as obtained with the entire gene set are marked with circles (*∘ *). The best overall results are highlighted in bold

Keyword Set	# Genes	TN	FP	FN	TP	Acc (%)
*Tuberculosis Data Set*						
*\emptyset *	25,345	8	2	2	8	80.0
$\{$’Diaphragm’$\}^{\circ }$	21	9	1	1	9	90.0
$\{$’DNA’*\}*	3,711	8	2	3	7	75.0
$\{$’Immune’*\}*	1,256	7	3	3	7	70.0
$\{$’Granulom’*\}*	1	9	1	5	5	70.0
$\{$’Lung’*\}*	186	8	2	5	5	65.0
$\{$’DNA Methylation’*\}*	38	6	4	3	7	65.0
$\{$’Immune System’*\}*	80	6	4	4	6	60.0
$\{$’Bacteria’*\}*	86	4	6	4	6	50.0
$\{$’TB’*\}*	29	6	4	6	4	50.0
– – – – – – – – – – – – – – – – – – – – – – – – – – – – – – – – – – – – – – – – – – – – – – – – – – – – – – – – – – – – –						
$\{$’Lung’, ’Immune System’$\}^{\circ }$	264	8	2	2	8	80.0
$\{$’TB’, ’Granulom’$\}^{\circ }$	30	9	1	3	7	80.0
$\{$’Diaphragm’, ’Bacteria’$\}^{\circ }$	107	8	2	2	8	80.0
$\{$’DNA’, ’Immune System’*\}*	3,752	8	2	3	7	75.0
$\{$’Bacteria’, ’Immune’, ’DNA’*\}*	4,679	8	2	3	7	75.0
– – – – – – – – – – – – – – – – – – – – – – – – – – – – – – – – – – – – – – – – – – – – – – – – – – – – – – – – – – – – –						
$\{$**’Diaphragm’**$\} \cap \{$**’DNA’***\}*	**4**	**9**	**1**	**0**	**10**	**95**.**0**
$\{$’Diaphragm’$\} \cap \{$’Lung’$\}^{\circ }$	2	8	2	2	8	80.0
$\{$’Lung’$\} \cap \{$’Immune System’*\}*	2	7	3	3	7	70.0
$\{$’Bacteria’$\} \cap \{$’DNA’*\}*	16	6	4	2	8	70.0
$\{$’TB’$\} \cap \{$’DNA’*\}*	25	8	2	4	6	70.0

*Prostate Cancer Data Set*						
*\emptyset *	25,603	16	10	11	16	60.4
$\{$’DNA’$\}^{\circ }$	3,749	17	9	10	17	64.2
$\{$’Immune’$\}^{\circ }$	1,276	17	9	10	17	64.2
$\{$’Estrogen’*\}*	184	15	11	11	16	58.5
$\{$’Prostate’*\}*	45	13	13	11	16	54.7
$\{$’Immune System’*\}*	77	13	13	13	14	50.9
$\{$’Testosterone’*\}*	74	12	14	13	14	49.1
– – – – – – – – – – – – – – – – – – – – – – – – – – – – – – – – – – – – – – – – – – – – – – – – – – – – – – – – – – – – –						
$\{$’Estrogen’, ’Prostate’$\}^{\circ }$	216	16	10	9	18	64.2
$\{$’Estrogen’, ’Immune’$\}^{\circ }$	1,442	17	9	10	17	64.2
– – – – – – – – – – – – – – – – – – – – – – – – – – – – – – – – – – – – – – – – – – – – – – – – – – – – – – – – – – – – –						
$\{$’Immune’$\} \cap \{$’Testosterone’*\}*	**7**	**14**	**12**	**5**	**22**	**67**.**9**
$\{$’DNA’$\} \cap \{$’Estrogen’$\}^{\circ }$	84	18	8	10	17	66.0
$\{$’Prostate’$\} \cap \{$’Estrogen’$\}^{\circ }$	13	15	11	9	18	62.3
$\{$’DNA’$\} \cap \{$’Immune’*\}*	304	15	11	15	12	50.9

**Table 14:** DNAm Data – Macro-averaged Accuracies in % (LOSO-CV with lin. **SVM Models**). *\emptyset *: Entire gene set. Keyword sets for which the MAA values were at least as high as obtained with the entire gene set are marked with circles (*∘ *). The best overall results are highlighted in bold

Keyword Set	# Genes	TN	FP	FN	TP	MAA (%)
*Breast Cancer Data Set*						
*\emptyset *	24,980	105	8	17	207	92.7
$\{$’Epithelial’*\}*	**761**	**106**	**7**	**14**	**210**	**93**.**8**
$\{$’Embryo’*\}*	740	103	10	15	209	92.2
$\{$’Cellular’*\}*	6,956	104	9	19	205	91.8
$\{$’Immune’*\}*	1,252	102	11	18	206	91.1
$\{$’DNA Methylation’*\}*	38	98	15	23	201	88.2
$\{$’Estrogen’*\}*	182	97	16	24	200	87.6
$\{$’Hormone’*\}*	609	97	16	22	202	88.0
– – – – – – – – – – – – – – – – – – – – – – – – – – – – – – – – – – – – – – – – – – – – – – – – – – – – – – – – – – – – –						
$\{$’Immune’, ’Epithelial’*\}*	1,930	104	9	17	207	92.2
$\{$’Cellular’, ’Immune’*\}*	7,361	104	9	19	205	91.8
$\{$’Cellular’, ’Epithelial’*\}*	7,151	103	10	18	206	91.6
– – – – – – – – – – – – – – – – – – – – – – – – – – – – – – – – – – – – – – – – – – – – – – – – – – – – – – – – – – – – –						
$\{$’Cellular’$\} \cap \{$’Embryo’$\}^{\circ }$	417	105	8	17	207	92.7
$\{$’Cellular’$\} \cap \{$’Immune’*\}*	847	105	8	19	205	92.2
$\{$’Embryo’$\} \cap \{$’Epithelial’*\}*	188	106	7	21	203	92.2
$\{$’Hormone’$\} \cap \{$’Estrogen’*\}*	49	105	8	21	203	91.8

*HCC Data Set*						
*\emptyset *	23,980	203	57	83	138	70.3
$\{$**’Protein’***\}*	**13,520**	**203**	**57**	**75**	**146**	**72**.**1**
$\{$’Cellular’*\}*	6,739	192	68	83	138	68.1
$\{$’Metabolic’*\}*	1,811	188	72	81	140	67.8
$\{$’DNA’*\}*	3,573	182	78	81	140	66.7
$\{$’DNA Methylation’*\}*	38	177	83	118	103	57.3
$\{$’Immune’*\}*	1,222	175	85	80	141	65.6
$\{$’Epithelial’*\}*	739	174	86	87	134	63.8
$\{$’Proliferation’*\}*	1,809	177	83	96	125	62.3
$\{$’Cytokine’*\}*	785	169	91	92	129	61.7
– – – – – – – – – – – – – – – – – – – – – – – – – – – – – – – – – – – – – – – – – – – – – – – – – – – – – – – – – – – – –						
$\{$’Cellular’, ’Epithelial’*\}*	6,927	196	64	81	140	69.4
$\{$’Cellular’, ’DNA Methylation’*\}*	6,762	173	87	88	133	68.6
$\{$’Cellular’, ’Immune’*\}*	7,135	190	70	82	139	68.0
– – – – – – – – – – – – – – – – – – – – – – – – – – – – – – – – – – – – – – – – – – – – – – – – – – – – – – – – – – – – –						
$\{$’Epithelial’$\} \cap \{$’Immune’*\}*	82	194	66	83	138	68.5
$\{$’Cellular’$\} \cap \{$’Protein’*\}*	5,967	188	72	85	136	66.9
$\{$’Protein’$\} \cap \{$’DNA’*\}*	3,067	187	73	87	134	66.3
$\{$’Metabolic’$\} \cap \{$’Proliferation’$\} \;\cap$	56	185	75	86	135	66.1
$$\{$$’Cytokine’*\}*

**Table 15:** RNA-Seq Data – Macro-averaged Accuracies in % (LOSO-CV with **LR Models**). *\emptyset *: Entire gene set. Keyword sets for which the MAA values were at least as high as obtained with the entire gene set are marked with circles (*∘ *). The best overall results are highlighted in bold

Keyword Set	# Genes	TN	FP	FN	TP	MAA (%)
*Thyroid Cancer Data Set*						
*\emptyset *	20,530	53	6	2	511	94.7
$\{$**’RNA’***\}*	**4,847**	**57**	**2**	**3**	**510**	**98**.**0**
$\{$’Thyroid’$\}^{\circ }$	133	57	2	5	508	97.8
$\{$’Epithelial’$\}^{\circ }$	745	57	2	6	507	97.7
$\{$’Thyroid’$\} \cap \{$’RNA’$\}^{\circ }$	76	55	4	6	507	96.0

*Liver Cancer Data Set*						
$\boldsymbol{\emptyset }$	**20,530**	**50**	**0**	**3**	**369**	**99**.**6**
$\{$’Gland’*\}*	358	50	0	4	368	99.5
$\{$’Blood’*\}*	712	50	0	4	368	99.5
$\{$’RNA’*\}*	4,847	50	0	4	368	99.5
$\{$’Lipid’*\}*	1,017	50	0	7	365	99.1
$\{$’Hepatocyte’*\}*	130	48	2	3	369	97.6
$\{$’Liver’*\}*	117	47	3	6	366	96.2
$\{$’Gland’$\} \cap \{$’RNA’*\}*	177	48	2	6	366	97.2

*Prostate Cancer Data Set*						
*\emptyset *	20,530	42	10	8	490	89.6
$\{$’RNA’$\}^{\circ }$	4,847	42	10	4	494	90.0
$\{$’Estrogen’*\}*	174	39	13	7	491	86.8
$\{$’Testosterone’*\}*	73	34	18	8	490	81.9
$\{$’Prostate’*\}*	45	30	22	13	485	77.5
$\{$**’Prostate’, ’RNA’***\}*	**4,862**	**43**	**9**	**4**	**494**	**90**.**9**
$\{$’Estrogen’, ’Testosterone’*\}*	226	38	14	7	491	85.8
$\{$’Estrogen’$\} \cap \{$’RNA’*\}*	81	36	16	6	492	84.0
$\{$’Prostate’$\} \cap \{$’RNA’*\}*	30	33	19	10	488	80.7
$\{$’Testosterone’$\} \cap \{$’RNA’*\}*	26	22	30	10	488	70.1

*Lung Cancer Data Set*						
*\emptyset *	20,530	108	2	4	985	98.9
$\{$**’RNA’***\}*	**4,847**	**108**	**2**	**2**	**987**	**99**.**0**
$\{$’Respiratory’*\}*	223	108	2	5	984	98.8
$\{$’Immune’*\}*	1,220	107	3	3	986	98.5
$\{$’Lung’*\}*	182	107	3	4	985	98.4
$\{$’Astrocyte’*\}*	108	107	3	8	981	98.2
$\{$’Trachea’*\}*	19	106	4	9	980	97.7
$\{$**’Respiratory’, ’Lung’***\}*	**388**	**108**	**2**	**2**	**987**	**99**.**0**
$\{$’Respiratory’, ’Trachea’*\}*	240	107	3	4	985	98.4
$\{$’Astrocyte’, ’Diaphragm’*\}*	129	107	3	6	983	98.3
$\{$’Respiratory’$\} \cap \{$’RNA’*\}*	42	107	3	6	983	98.3
$\{$’Trachea’$\} \cap \{$’RNA’*\}*	13	107	3	10	979	98.1
$\{$’Lung’$\} \cap \{$’RNA’*\}*	100	106	4	7	982	97.8

This table depicts the results for the four Kaggle data sets

**Table 16:** RNA-Seq Data – Macro-averaged Accuracies in % (LOSO-CV with **LR Models**). *\emptyset *: Entire gene set. The best overall results are highlighted in bold

Keyword Set	# Genes	TN	FP	FN	TP	MAA (%)
*Leukaemia Data Set*						
*\emptyset *	35,087	8	0	1	64	99.2
$\{$**’Lymph’***\}*	**149**	**8**	**0**	**0**	**65**	**100**
$\{$**’Blood’***\}*	**772**	**8**	**0**	**0**	**65**	**100**
$\{$**’RNA’***\}*	**5,358**	**8**	**0**	**0**	**65**	**100**
$\{$**’Plasma’***\}*	**5,685**	**8**	**0**	**0**	**65**	**100**

*Liver Disease Data Set*						
$\boldsymbol{\emptyset }$	**40,131**	**26**	**1**	**0**	**216**	**98**.**1**
$\{$**’Gland’***\}*	**364**	**26**	**1**	**0**	**216**	**98**.**1**
$\{$**’Lipid’***\}*	**1,134**	**26**	**1**	**0**	**216**	**98**.**1**
$\{$’Hepatocyte’*\}*	130	26	1	1	215	97.9
$\{$’Blood’*\}*	768	26	1	1	215	97.9
$\{$’RNA’*\}*	5,334	26	1	1	215	97.9
$\{$’Liver’*\}*	117	26	1	2	214	97.7

This table depicts the results for the two GEO data sets

**Table 17:** DNAm Data – Accuracies (Acc) in % (LOSO-CV with **LR Models**).*\emptyset *: Entire gene set. Keyword sets for which the Acc values were at least as high as obtained with the entire gene set are marked with circles (*∘ *). The best overall results are highlighted in bold

Keyword Set	# Genes	TN	FP	FN	TP	Acc (%)
*Tuberculosis Data Set*						
*\emptyset *	25,345	8	2	5	5	65.0
$\{$**’Lung’***\}*	**186**	**9**	**1**	**1**	**9**	**90**.**0**
$\{$’Diaphragm’$\}^{\circ }$	21	8	2	1	9	85.0
$\{$’DNA Methylation’$\}^{\circ }$	38	9	1	4	6	75.0
$\{$’Bacteria’$\}^{\circ }$	86	8	2	3	7	75.0
$\{$’Granulom’$\}^{\circ }$	1	9	1	5	5	70.0
$\{$’Immune System’$\}^{\circ }$	80	8	2	4	6	70.0
$\{$’Immune’$\}^{\circ }$	1,256	8	2	4	6	70.0
$\{$’DNA’*\}*	3,711	7	3	5	5	60.0
$\{$’TB’*\}*	29	6	4	8	2	40.0
– – – – – – – – – – – – – – – – – – – – – – – – – – – – – – – – – – – – – – – – – – – – – – – – – – – – – – – – – – – – –						
$\{$’Diaphragm’, ’Bacteria’$\}^{\circ }$	107	9	1	3	7	80.0
$\{$’Lung’, ’Immune System’$\}^{\circ }$	264	8	2	3	7	75.0
$\{$’Bacteria’, ’Immune’, ’DNA’$\}^{\circ }$	4,679	8	2	4	6	70.0
$\{$’DNA’, ’Immune System’$\}^{\circ }$	3,752	7	3	4	6	65.0
$\{$’TB’, ’Granulom’*\}*	30	6	4	6	4	50.0
– – – – – – – – – – – – – – – – – – – – – – – – – – – – – – – – – – – – – – – – – – – – – – – – – – – – – – – – – – – – –						
$\{$**’Diaphragm’**$\} \cap \{$**’DNA’***\}*	**4**	**9**	**1**	**1**	**9**	**90**.**0**
$\{$’Diaphragm’$\} \cap \{$’Lung’$\}^{\circ }$	2	7	3	2	8	75.0
$\{$’Lung’$\} \cap \{$’Immune System’$\}^{\circ }$	2	7	3	3	7	70.0
$\{$’Bacteria’$\} \cap \{$’DNA’$\}^{\circ }$	16	6	4	2	8	70.0
$\{$’TB’$\} \cap \{$’DNA’*\}*	25	5	5	5	5	50.0

*Prostate Cancer Data Set*						
*\emptyset *	25,603	15	11	9	18	62.3
$\{$’Immune System’$\}^{\circ }$	77	19	7	12	15	64.2
$\{$’Testosterone’$\}^{\circ }$	74	16	10	10	17	62.3
$\{$’Immune’*\}*	1,276	15	11	12	15	56.6
$\{$’DNA’*\}*	3,749	16	10	13	14	56.6
$\{$’Estrogen’*\}*	184	15	11	15	12	50.9
$\{$’Prostate’*\}*	45	16	10	23	4	37.7
– – – – – – – – – – – – – – – – – – – – – – – – – – – – – – – – – – – – – – – – – – – – – – – – – – – – – – – – – – – – –						
$\{$’Estrogen’, ’Immune’*\}*	1,442	16	10	12	15	58.5
$\{$’Estrogen’, ’Prostate’*\}*	216	14	12	12	15	54.7
– – – – – – – – – – – – – – – – – – – – – – – – – – – – – – – – – – – – – – – – – – – – – – – – – – – – – – – – – – – – –						
$\{$**’DNA’**$\} \cap \{$**’Immune’***\}*	**304**	**19**	**7**	**10**	**17**	**67**.**9**
$\{$’Prostate’$\} \cap \{$’Estrogen’*\}*	13	14	12	10	17	58.5
$\{$’DNA’$\} \cap \{$’Estrogen’*\}*	84	15	11	11	16	58.5
$\{$’Immune’$\} \cap \{$’Testosterone’*\}*	7	16	10	16	11	50.9

**Table 18:** DNAm Data – Macro-averaged Accuracies in % (LOSO-CV with **LR Models**). *\emptyset *: Entire gene set. Keyword sets for which the MAA values were at least as high as obtained with the entire gene set are marked with circles (*∘ *). The best overall results are highlighted in bold

Keyword Set	# Genes	TN	FP	FN	TP	MAA (%)
*Breast Cancer Data Set*						
*\emptyset *	24,980	107	6	29	195	90.9
$\{$**’Epithelial’***\}*	**761**	**105**	**8**	**14**	**210**	**93**.**3**
$\{$’Cellular’$\}^{\circ }$	6,956	102	11	15	209	91.8
$\{$’Hormone’$\}^{\circ }$	609	102	11	19	205	90.9
$\{$’Embryo’$\}^{\circ }$	740	103	10	21	203	90.9
$\{$’Immune’$\}^{\circ }$	1,252	104	9	23	201	90.9
$\{$’Estrogen’*\}*	182	104	9	24	200	90.7
$\{$’DNA Methylation’*\}*	38	99	14	21	203	89.1
– – – – – – – – – – – – – – – – – – – – – – – – – – – – – – – – – – – – – – – – – – – – – – – – – – – – – – – – – – – – –						
$\{$’Immune’, ’Epithelial’$\}^{\circ }$	1,930	104	9	16	208	92.4
$\{$’Cellular’, ’Immune’$\}^{\circ }$	7,361	103	10	16	208	92.0
$\{$’Cellular’, ’Epithelial’$\}^{\circ }$	7,151	102	11	15	209	91.8
– – – – – – – – – – – – – – – – – – – – – – – – – – – – – – – – – – – – – – – – – – – – – – – – – – – – – – – – – – – – –						
$\{$’Cellular’$\} \cap \{$’Embryo’$\}^{\circ }$	417	105	8	20	204	92.0
$\{$’Embryo’$\} \cap \{$’Epithelial’*\}*	188	102	11	21	203	90.4
$\{$’Hormone’$\} \cap \{$’Estrogen’*\}*	49	101	12	23	201	89.6
$\{$’Cellular’$\} \cap \{$’Immune’*\}*	847	101	12	24	200	89.3

*HCC Data Set*						
*\emptyset *	23,980	178	82	84	137	65.2
$\{$’DNA’$\}^{\circ }$	3,573	182	78	76	145	67.8
$\{$’Protein’$\}^{\circ }$	13,520	196	64	91	130	67.1
$\{$’Cellular’$\}^{\circ }$	6,739	179	81	80	141	66.3
$\{$’Cytokine’*\}*	785	173	87	82	139	64.7
$\{$’Metabolic’*\}*	1,811	173	87	84	137	64.3
$\{$’Immune’*\}*	1,222	170	90	87	134	63.0
$\{$’Proliferation’*\}*	1,809	179	81	97	124	62.5
$\{$’Epithelial’*\}*	739	164	96	87	134	61.9
$\{$’DNA Methylation’*\}*	38	146	114	90	131	57.7
– – – – – – – – – – – – – – – – – – – – – – – – – – – – – – – – – – – – – – – – – – – – – – – – – – – – – – – – – – – – –						
$\{$**’Cellular’, ’Epithelial’***\}*	**6,927**	**194**	**66**	**83**	**138**	**68**.**5**
$\{$’Cellular’, ’Immune’$\}^{\circ }$	7,135	183	77	80	141	67.1
$\{$’Cellular’, ’DNA Methylation’$\}^{\circ }$	6,762	188	72	86	135	66.7
– – – – – – – – – – – – – – – – – – – – – – – – – – – – – – – – – – – – – – – – – – – – – – – – – – – – – – – – – – – – –						
$\{$’Epithelial’$\} \cap \{$’Immune’$\}^{\circ }$	82	180	80	82	139	66.1
$\{$’Protein’$\} \cap \{$’DNA’$\}^{\circ }$	3,067	179	81	83	138	65.6
$\{$’Cellular’$\} \cap \{$’Protein’$\}^{\circ }$	5,967	186	74	89	132	65.6
$\{$’Metabolic’$\} \cap \{$’Proliferation’$\} \;\cap$	56	158	102	74	147	63.6
$$\{$$’Cytokine’*\}*

**Table 19:** RNA-Seq Data – Macro-averaged Accuracies in % (LOSO-CV with **RF Models**).*\emptyset *: Entire gene set. Keyword sets for which the MAA values were at least as high as obtained with the entire gene set are marked with circles (*∘ *). The best overall results are highlighted in bold

Keyword Set	# Genes	TN	FP	FN	TP	MAA (%)
*Thyroid Cancer Data Set*						
$\boldsymbol{\emptyset }$	**20,530**	**53**	**6**	**2**	**511**	**94**.**7**
$\{$’Epithelial’*\}*	745	52	7	2	511	93.9
$\{$’Thyroid’*\}*	133	50	9	1	512	92.3
$\{$’RNA’*\}*	4,847	47	12	2	511	89.6
$\{$’Thyroid’$\} \cap \{$’RNA’*\}*	76	47	12	2	511	89.6

*Liver Cancer Data Set*						
*\emptyset *	20,530	47	3	4	368	96.5
$\{$**’Blood’***\}*	**712**	**48**	**2**	**3**	**369**	**97**.**6**
$\{$’RNA’*\}*	4,847	47	3	5	367	96.3
$\{$’Lipid’*\}*	1,017	44	6	2	370	93.7
$\{$’Gland’*\}*	358	44	6	4	368	93.5
$\{$’Hepatocyte’*\}*	130	43	7	2	370	92.7
$\{$’Liver’*\}*	117	40	10	3	369	89.6
$\{$’Gland’$\} \cap \{$’RNA’*\}*	177	42	8	4	368	91.5

*Prostate Cancer Data Set*						
*\emptyset *	20,530	30	22	7	491	78.1
$\{$’RNA’$\}^{\circ }$	4,847	30	22	5	493	78.3
$\{$’Prostate’*\}*	45	29	23	7	491	77.2
$\{$’Estrogen’*\}*	174	28	24	6	492	76.3
$\{$’Testosterone’*\}*	73	26	26	6	492	74.4
$\{$**’Prostate’, ’RNA’***\}*	**4,862**	**31**	**21**	**5**	**493**	**79**.**3**
$\{$’Estrogen’, ’Testosterone’*\}*	226	29	23	4	494	77.5
$\{$’Estrogen’$\} \cap \{$’RNA’$\}^{\circ }$	81	31	21	6	492	79.2
$\{$’Prostate’$\} \cap \{$’RNA’*\}*	30	30	22	10	488	77.8
$\{$’Testosterone’$\} \cap \{$’RNA’*\}*	26	25	27	8	490	73.2

*Lung Cancer Data Set*						
*\emptyset *	20,530	107	3	3	986	98.5
$\{$**’Immune’***\}*	**1,220**	**109**	**1**	**4**	**985**	**99**.**3**
$\{$’Respiratory’$\}^{\circ }$	223	108	2	5	984	98.8
$\{$’Astrocyte’$\}^{\circ }$	108	107	3	3	986	98.5
$\{$’RNA’$\}^{\circ }$	4,847	107	3	3	986	98.5
$\{$’Lung’*\}*	182	105	5	5	984	97.5
$\{$’Trachea’*\}*	19	98	12	5	984	94.3
$\{$**’Respiratory’, ’Trachea’***\}*	**240**	**109**	**1**	**4**	**985**	**99**.**3**
$\{$’Astrocyte’, ’Diaphragm’$\}^{\circ }$	129	108	2	3	986	98.9
$\{$’Respiratory’, ’Lung’$\}^{\circ }$	388	108	2	4	985	98.9
$\{$’Lung’$\} \cap \{$’RNA’*\}*	100	102	8	6	983	96.1
$\{$’Respiratory’$\} \cap \{$’RNA’*\}*	42	100	10	7	982	95.1
$\{$’Trachea’$\} \cap \{$’RNA’*\}*	13	99	11	6	983	94.7

This table depicts the results for the four Kaggle data sets

**Table 20:** RNA-Seq Data – Macro-averaged Accuracies in % (LOSO-CV with **RF Models**). *\emptyset *: Entire gene set. The best overall results are highlighted in bold

Keyword Set	# Genes	TN	FP	FN	TP	MAA (%)
*Leukaemia Data Set*						
$\boldsymbol{\emptyset }$	**35,087**	**8**	**0**	**0**	**65**	**100**
$\{$**’Lymph’***\}*	**149**	**8**	**0**	**0**	**65**	**100**
$\{$**’Blood’***\}*	**772**	**8**	**0**	**0**	**65**	**100**
$\{$**’RNA’***\}*	**5,358**	**8**	**0**	**0**	**65**	**100**
$\{$**’Plasma’***\}*	**5,685**	**8**	**0**	**0**	**65**	**100**

*Liver Disease Data Set*						
*\emptyset *	40,131	27	0	2	214	99.5
$\{$**’Blood’***\}*	**768**	**27**	**0**	**1**	**215**	**99**.**8**
$\{$’Liver’*\}*	117	26	1	1	215	97.9
$\{$’Gland’*\}*	358	26	1	1	215	97.9
$\{$’RNA’*\}*	5,334	26	1	1	215	97.9
$\{$’Hepatocyte’*\}*	130	26	1	2	214	97.7
$\{$’Lipid’*\}*	1,134	26	1	2	214	97.7

This table depicts the results for the two GEO data sets

**Table 21:** DNAm Data – Accuracies (Acc) in % (LOSO-CV with **RF Models**). *\emptyset *: Entire gene set. Keyword sets for which the Acc values were at least as high as obtained with the entire gene set are marked with circles (*∘ *). The best overall results are highlighted in bold

Keyword Set	# Genes	TN	FP	FN	TP	Acc (%)
*Tuberculosis Data Set*						
*\emptyset *	25,345	8	2	6	4	60.0
$\{$**’Diaphragm’***\}*	**21**	**9**	**1**	**2**	**8**	**85**.**0**
$\{$’Bacteria’$\}^{\circ }$	86	9	1	3	7	80.0
$\{$’DNA Methylation’$\}^{\circ }$	38	8	2	3	7	75.0
$\{$’Immune’$\}^{\circ }$	1,256	8	2	4	6	70.0
$\{$’Lung’$\}^{\circ }$	186	7	3	4	6	65.0
$\{$’Immune System’$\}^{\circ }$	80	6	4	4	6	60.0
$\{$’DNA’$\}^{\circ }$	3,711	7	3	5	5	60.0
$\{$’Granulom’*\}*	1	6	4	5	5	55.0
$\{$’TB’*\}*	29	5	5	5	5	50.0
– – – – – – – – – – – – – – – – – – – – – – – – – – – – – – – – – – – – – – – – – – – – – – – – – – – – – – – – – – – – –						
$\{$’Diaphragm’, ’Bacteria’$\}^{\circ }$	107	9	1	3	7	80.0
$\{$’Lung’, ’Immune System’$\}^{\circ }$	264	8	2	4	6	70.0
$\{$’DNA’, ’Immune System’$\}^{\circ }$	3,752	9	1	5	5	70.0
$\{$’Bacteria’, ’Immune’, ’DNA’$\}^{\circ }$	4,679	7	3	4	6	65.0
$\{$’TB’, ’Granulom’*\}*	30	7	3	6	4	55.0
– – – – – – – – – – – – – – – – – – – – – – – – – – – – – – – – – – – – – – – – – – – – – – – – – – – – – – – – – – – – –						
$\{$’Diaphragm’$\} \cap \{$’DNA’$\}^{\circ }$	4	8	2	2	8	80.0
$\{$’Diaphragm’$\} \cap \{$’Lung’$\}^{\circ }$	2	7	3	4	6	65.0
$\{$’TB’$\} \cap \{$’DNA’$\}^{\circ }$	25	7	3	4	6	65.0
$\{$’Lung’$\} \cap \{$’Immune System’$\}^{\circ }$	2	6	4	4	6	60.0
$\{$’Bacteria’$\} \cap \{$’DNA’*\}*	16	5	5	5	5	50.0

*Prostate Cancer Data Set*						
*\emptyset *	25,603	13	13	13	14	50.9
$\{$’Testosterone’$\}^{\circ }$	74	17	9	12	15	60.4
$\{$’Prostate’$\}^{\circ }$	45	17	9	13	14	58.5
$\{$’Immune’$\}^{\circ }$	1,276	13	13	10	17	56.6
$\{$’Immune System’$\}^{\circ }$	77	12	14	10	17	54.7
$\{$’DNA’$\}^{\circ }$	3,749	13	13	11	16	54.7
$\{$’Estrogen’*\}*	184	12	14	13	14	49.1
– – – – – – – – – – – – – – – – – – – – – – – – – – – – – – – – – – – – – – – – – – – – – – – – – – – – – – – – – – – – –						
$\{$’Estrogen’, ’Prostate’$\}^{\circ }$	216	13	13	10	17	56.6
$\{$’Estrogen’, ’Immune’$\}^{\circ }$	1,442	13	13	13	14	50.9
– – – – – – – – – – – – – – – – – – – – – – – – – – – – – – – – – – – – – – – – – – – – – – – – – – – – – – – – – – – – –						
$\{$**’DNA’**$\} \cap \{$**’Immune’***\}*	**304**	**17**	**9**	**11**	**16**	**62**.**3**
$\{$’Immune’$\} \cap \{$’Testosterone’$\}^{\circ }$	7	14	12	11	16	56.6
$\{$’DNA’$\} \cap \{$’Estrogen’$\}^{\circ }$	84	13	13	12	15	52.8
$\{$’Prostate’$\} \cap \{$’Estrogen’*\}*	13	9	17	16	11	37.7

**Table 22:** DNAm Data – Macro-averaged Accuracies in % (LOSO-CV with **RF Models**). *\emptyset *: Entire gene set. The best overall results are highlighted in bold

Keyword Set	# Genes	TN	FP	FN	TP	MAA (%)
*Breast Cancer Data Set*						
$\boldsymbol{\emptyset }$	**24,980**	**100**	**13**	**16**	**208**	**90**.**7**
$\{$’Estrogen’*\}*	182	99	14	15	209	90.5
$\{$’Epithelial’*\}*	761	99	14	19	205	89.6
$\{$’Cellular’*\}*	6,956	97	16	16	208	89.3
$\{$’Hormone’*\}*	609	97	16	17	207	89.1
$\{$’Immune’*\}*	1,252	97	16	17	207	89.1
$\{$’Embryo’*\}*	740	97	16	19	205	88.7
$\{$’DNA Methylation’*\}*	38	94	19	19	205	87.4
– – – – – – – – – – – – – – – – – – – – – – – – – – – – – – – – – – – – – – – – – – – – – – – – – – – – – – – – – – – – –						
$\{$’Cellular’, ’Immune’*\}*	7,361	98	15	17	207	89.6
$\{$’Immune’, ’Epithelial’*\}*	1,930	97	16	17	207	89.1
$\{$’Cellular’, ’Epithelial’*\}*	7,151	97	16	17	207	89.1
– – – – – – – – – – – – – – – – – – – – – – – – – – – – – – – – – – – – – – – – – – – – – – – – – – – – – – – – – – – – –						
$\{$’Embryo’$\} \cap \{$’Epithelial’*\}*	188	99	14	16	208	90.2
$\{$’Cellular’$\} \cap \{$’Embryo’*\}*	417	98	15	20	204	88.9
$\{$’Cellular’$\} \cap \{$’Immune’*\}*	847	95	18	18	206	88.0
$\{$’Hormone’$\} \cap \{$’Estrogen’*\}*	49	93	20	18	206	87.1

*HCC Data Set*						
$\boldsymbol{\emptyset }$	**23,980**	**206**	**54**	**103**	**118**	**66**.**3**
$\{$’Epithelial’*\}*	739	198	62	100	121	65.5
$\{$’DNA’*\}*	3,573	204	56	110	111	64.3
$\{$’Cellular’*\}*	6,739	202	58	109	112	64.2
$\{$’Protein’*\}*	13,520	203	57	110	111	64.2
$\{$’Proliferation’*\}*	1,809	202	58	110	111	64.0
$\{$’Cytokine’*\}*	785	192	68	102	119	63.8
$\{$’Metabolic’*\}*	1,811	200	60	110	111	63.6
$\{$’Immune’*\}*	1,222	197	63	112	109	62.5
$\{$’DNA Methylation’*\}*	38	191	69	129	92	57.5
– – – – – – – – – – – – – – – – – – – – – – – – – – – – – – – – – – – – – – – – – – – – – – – – – – – – – – – – – – – – –						
$\{$’Cellular’, ’DNA Methylation’*\}*	6,762	207	53	110	111	64.9
$\{$’Cellular’, ’Immune’*\}*	7,135	195	65	107	114	63.3
$\{$’Cellular’, ’Epithelial’*\}*	6,927	193	67	114	107	61.3
– – – – – – – – – – – – – – – – – – – – – – – – – – – – – – – – – – – – – – – – – – – – – – – – – – – – – – – – – – – – –						
$\{$’Metabolic’$\} \cap \{$’Proliferation’$\} \;\cap$	56	204	56	102	119	66.2
$\{$’Cytokine’*\}*						
$\{$’Cellular’$\} \cap \{$’Protein’*\}*	5,967	204	56	106	115	65.2
$\{$’Epithelial’$\} \cap \{$’Immune’*\}*	82	193	67	109	112	62.5
$\{$’Protein’$\} \cap \{$’DNA’*\}*	3,067	197	63	112	109	62.5

## Footnotes

1 Website: https://geneontology.org (Last accessed on March 9, 2026).

2 Website: https://www.ncbi.nlm.nih.gov/geo/ (Last accessed on March 9, 2026).

3 Website: https://www.kaggle.com (Last accessed on March 9, 2026).

4 For details, see website https://www.ensembl.org/index.html (Last accessed on March 9, 2026).
